# Significantly Improved HIV Inhibitor Efficacy Prediction Employing Proteochemometric Models Generated From Antivirogram Data

**DOI:** 10.1371/journal.pcbi.1002899

**Published:** 2013-02-21

**Authors:** Gerard J. P. van Westen, Alwin Hendriks, Jörg K. Wegner, Adriaan P. IJzerman, Herman W. T. van Vlijmen, Andreas Bender

**Affiliations:** 1 Division of Medicinal Chemistry, Leiden/Amsterdam Center for Drug Research, Leiden, The Netherlands; 2 Tibotec BVBA, Beerse, Belgium; 3 Unilever Centre for Molecular Science Informatics, Department of Chemistry, University of Cambridge, Cambridge, United Kingdom; University of California San Diego, United States of America

## Abstract

Infection with HIV cannot currently be cured; however it can be controlled by combination treatment with multiple anti-retroviral drugs. Given different viral genotypes for virtually each individual patient, the question now arises which drug combination to use to achieve effective treatment. With the availability of viral genotypic data and clinical phenotypic data, it has become possible to create computational models able to predict an optimal treatment regimen for an *individual* patient. Current models are based only on sequence data derived from viral genotyping; chemical similarity of drugs is not considered. To explore the added value of chemical similarity inclusion we applied proteochemometric models, combining chemical and protein target properties in a single bioactivity model. Our dataset was a large scale clinical database of genotypic and phenotypic information (in total ca. 300,000 drug-mutant bioactivity data points, 4 (NNRTI), 8 (NRTI) or 9 (PI) drugs, and 10,700 (NNRTI) 10,500 (NRTI) or 27,000 (PI) mutants). Our models achieved a prediction error below 0.5 Log Fold Change. Moreover, when directly compared with previously published sequence data, derived models PCM performed better in resistance classification and prediction of Log Fold Change (0.76 log units versus 0.91). Furthermore, we were able to successfully confirm both known and identify *previously unpublished*, resistance-conferring mutations of HIV Reverse Transcriptase (e.g. K102Y, T216M) and HIV Protease (e.g. Q18N, N88G) from our dataset. Finally, we applied our models prospectively to the public HIV resistance database from Stanford University obtaining a correct resistance prediction rate of 84% on the full set (compared to 80% in previous work on a *high quality subset*). We conclude that proteochemometric models are able to accurately predict the phenotypic resistance based on genotypic data even for novel mutants *and mixtures*. Furthermore, we add an applicability domain to the prediction, informing the user about the reliability of predictions.

## Author Summary

Infection with the human immunodeficiency virus (HIV) currently cannot be cured. It can however be contained through treatment with a combination of several anti-viral drugs. Yet, during treatment resistance can occur which leads to drugs becoming ineffective. Through a combination of drugs, this resistance can be deferred indefinitely. The optimal combination of drugs depends on the specific strain of HIV with which the patient is infected. Previously, methods have been developed that predict a personalized treatment regimen based on the genetic sequence (genotype) of the virus via the use of computer modeling, corner stone of the methods is drug affinity prediction. Here we have applied proteochemometric modeling which takes this genetic information into account, but also includes chemical description of the drugs that are now clinically available. We show that this combined technique performs better than models that only include genetic information. Our approach leads to personalized treatment predictions with a higher reliability compared to the current state of the art. In addition, we include a reliability measure which allows each prediction to be assessed for reliability. Finally we describe mutations of the HIV genome that were not previously described in literature and lead to resistance to treatment.

## Introduction

The Human Immunodeficiency Virus (HIV) was discovered and isolated as the cause of ‘Acquired Immuno Deficiency Syndrome’ (AIDS) in 1983. [1], [2] Over the following three decades HIV has turned into a global epidemic, the number of people living with HIV in 2010 being estimated at 34 million according to the World Health Organization. [3] Furthermore the number of people newly infected was approximately 2.7 million and 1.8 million HIV related deaths were reported, [3] hence illustrating that HIV represents one of the major illnesses of mankind today.

Infection with HIV can be contained, however not cured, by Highly Active Anti-Retroviral Therapy (HAART), which relies on a combination of three or more inhibitors from different drug classes. [4], [5] Currently more than 20 HIV inhibiting dugs are approved, [6] with the largest classes of drugs being formed by Protease Inhibitors (PIs), Nucleoside/Nucleotide Reverse Transcriptase Inhibitors (NRTIs) and Non-Nucleoside Reverse Transcriptase Inhibitors (NNRTIs). However, while a large number of drugs is accessible to the physician (thus rendering HIV in some sense a disease that is currently ‘under control’ regarding the treatment options available), the question of *which drugs to use for which patient* is an exercise where more guidance would also in the current situation be of tremendous practical relevance.

### Genetic variability

The process of replication by HIV is extremely error prone and therefore mutations in the viral genome occur frequently. [7], [8] It is these mutations that can be the basis for HIV resistance against therapy, [6] even single point mutations can cause insensitivity of HIV to treatment with all members from an entire drug class (e.g. K101P in the case of NNRTIs). [6], [9] Occurrence of these resistance conferring mutations can be contained or minimized by the nature of HAART therapy due to the combination of multiple drugs classes. [5] However, the occurrence of high impact mutations can cause treatment failure in HAART for certain specific drug regimens. It is therefore crucial that the drug regimen is tailored to the specific viral genotype. [10], [11]

### Personalized medicine

What is required for a tailored drug regimen is knowledge of the effect of individual mutations on the efficacy of different drugs. A rough distinction can be made between assay based methods and computational methods, with assay based methods being available since the year 1998. [12], [13], [14] Conversely, various computational methods have become available over the last decade. [15], [16], [17], [18], [19], [20] Personalized prediction has been shown to perform equal to standard of care in treatment naïve patients but significantly (P = 0.02) better in patients experiencing drug failure. [17] Furthermore, computational approaches have been shown to perform equal to phenotypic assays. [21] Several methods that have been published previously, both assay-based and computational approaches, will be outlined briefly in the following.

### Phenotypic assays

Phenotypic assays measure the replication of HIV *in vitro* subsequent to genotype determination. Three common different phenotypic assays include: Antivirogram (AVG) by Virco (1998), [12] an assay by Walter *et al.* by the Universities of Erlangen-Nürnberg and Leuven (1999), [14] and Phenosense by Monogram Biosciences (2000). [13] Diverse readouts are employed in these assays: spectrophotometrical determination of diphenyltetrazolium bromide reduction (AVG), luminescence produced by secreted alkaline phosphatase (Walter *et al.*), [14] and luminescence by luciferase produced in the cell upon completion of one round of virus replication (Phenosense). All readouts respond in a dose dependent manner. Antiretroviral drug susceptibility is expressed as the base 10 logarithm of a numerical fold change (Log FC). Log FC is determined by dividing the IC_50_ for inhibition of the mutated virus by the IC_50_ for inhibition of a determined wild type virus (wt). Hence, a Log FC value of 1 for a given drug – mutant pair means that the drug IC_50_ value for that particular mutant is 10 times that of the IC_50_ value for the same drug on wt. Likewise, a Log FC value of 3 for a given drug – mutant pair represents an IC_50_ value 1,000 times higher. The sequences that are defined to be wt are the HXB2 strain (Uniprot accession P04585) for AVG, [22], [23] or a recombinant pNL4-3 strain (Genbank entry M19921) for Walter *et al.* and PhenoSense. [24]

### Virtual phenotype approaches

From the data generated by the phenotypic assays, computational models have been produced that predict a virtual phenotype from a given genotype. Based on the large amount of Log FC data generated by AVG, Virco introduced their first computational prediction tool, Virtual Phenotype in 2000 superseded by VircoTYPE HIV-1 in 2004. [25] VircoTYPE creates linear regression models based on the presence of mutations and pairs of mutations. Each mutation and mutation pair is given a weight factor in model training based on measured data (6,000 to 40,000 samples per drug). The sum of all weight factors for relevant mutations present in a mutant combined with the wild type weight factor then provides the predicted log FC. In a randomized clinical trial, VircoTYPE HIV-1 has been shown to perform slightly better than conventional phenotypic assays in decreasing HIV RNA concentration over a follow up period of 48 weeks (39% of the phenotypic assay group reached HIV RNA below 400 copies/ml compared to 51% of the VircoTYPE HIV-1 group). [21]

Next to VircoTYPE HIV-1, another implementation of a virtual phenotype has been developed at the Max Planck Institute, called Geno2Pheno. [20] This tool has been trained on smaller dataset compared to VircoTYPE. However, it has been retrospectively validated on the Stanford HIV Drug Resistance Database (Stanford Set) in 2009. [19] In this study Geno2Pheno outperformed state-of-the-art-expert based systems by finding 16.2–19.8% more successful regimens.

Nevertheless, what the computational methods described here have in common is that they are solely trained on the mutation patterns and the effect these patterns have on a *single* drug. [26], [27], [28] Therefore a separate model is created for every drug. Similarity between individual amino acids is not considered (how similar are two amino acids to each other and hence how big is the impact of a mutation). Furthermore, the chemical similarity between compounds is not considered in the models. Both types of similarity information have the potential to lead to better models and prompted us to apply ‘proteochemometric models’, described in the following, to improve upon the current situation.

### Proteochemometric modeling

**Figure 1:**
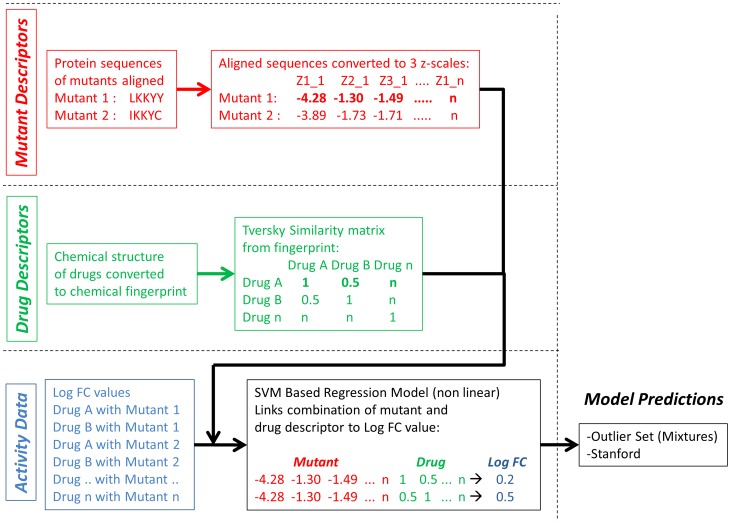
Flowchart of the work performed here. A distinction is made between the preparation of mutant descriptors (shown in red), drug descriptors (shown in green) and activity data (Log FC, shown in blue). The descriptors and Log FC values are combined, and subsequently a model is created using a non-linear modeling technique (support vector machines, SVM). The final step is model validation (shown in black) which is done both on the outlier set by van der Borght et al. and the independent Stanford university set.

Given that previous models did not take into account chemical information, the individual models mentioned above fail to acknowledge the chemical similarity between drugs that belong to a single class, thereby discarding very valuable information. This is the case because molecular similarity has been shown to have great predictive power when it comes to identifying which kind of *related* structures could also show activity against a given target. [29] Hence it is likely that also for established drugs, chemical similarity can improve models by explicitly taking the concept of drug – target interaction into account, which is then combined with mutational information of the drug target itself. This technique is called proteochemometric (PCM) modeling. The concept of PCM as we applied it has been summarized in Figure 1. This flow chart shows how we combine both mutant data and drug data and link it to a Log FC value. The authors have previously reviewed the technique and it has already been successfully applied to NNRTI inhibitors of HIV Reverse Transcriptase before. [30], [31], [32], [33]

Yet, the most important difference between this previous work and the current study is the *scale* of the mutant database used to train the models on. Previous work focused on a total of 4,792 data points, [30] 386 data points, [34] 654 data points, [31] 4,495 data points, [35] or 4,024 data points, [33] whereas here a total of 288,138 data points are used. Hence, we expect a more generally applicable model resulting from the current study. Furthermore, previous work included a larger number of compounds (451 compounds) on the chemical side, and their biological activity on a total of 14 mutants. Therefore, these models described a relatively large chemical space compared to the target space, while in the current work we have reversed this situation and the models now describe a relatively large target space compared (approximately 37,000 mutants) to the chemical space (21 drugs). In addition, what is lacking in previously published PCM approaches is the power to extrapolate, thereby able to also produce a reliable prediction for novel (unknown) mutants while including a reliability measure for these predictions. These are the points we are addressing in the current work.

### Aim of the project

In the current project it is our hypothesis that we can train a single PCM model for each of the following major HIV drug classes using the AVG data: Protease Inhibitors (PIs), Nucleoside/Nucleotide Reverse Transcriptase Inhibitors (NRTIs) and Non-Nucleoside Reverse Transcriptase Inhibitors (NNRTIs). As no PCM model has ever been trained on such a large dataset (the current dataset is 60 times larger than the largest published HIV PCM model) our hypothesis was on the one hand to arrive at better model performance, and on the other hand to unravel more reliable rules such as the influence of point mutations on compound activity. Scientifically interesting is also the reversal in the ratio between chemical space and target space in the model training set described above.

Given the wealth of training data present, the resulting bioactivity models can be used to predict the activity of clinical ARV drugs on mutants *not present* (untested) in the dataset (corresponding to a patient with a new, previously unseen genotype that needs to be treated in the clinic). For this purpose, an additional 7,798 data points have been used as a *prospective validation set*, in order to gauge predictive performance of the model in a real-world situation. These data points have been retrieved from the Stanford University database after model training and validation was completed.

## Results/Discussion

### PCM model validation (internal)

We first validated by creating a learning curve for each drug class. Learning curves plot the quality of models that are created on an increasing fraction of the data. Concurrently these models are validated on the remainder (and hence decreasing) part of the dataset (supporting Figure S1). When given enough measures of model reliability in respect to the training set size, an estimate can be made of the optimal performance possible on said dataset.

We found that all models should reach a root mean square error (RMSE) <0.5 units Log FC (see Methods section and supporting Figure S1), which was subsequently confirmed in the external validation which was performed per drug rather than per drug class below (See supporting Table S1 for the used abbreviations for each drug).

### PCM model validation (external)

**Figure 2:**
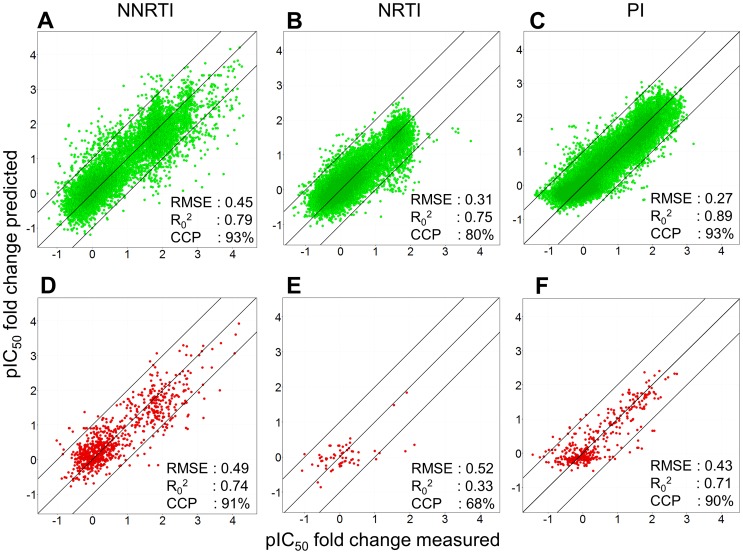
Model validation. (A,B,C) Our models perform robustly in both internal validation (unknown combinations of known drugs and known mutants) and (D,E,F) external validation (unknown combinations of drugs and mutants, one of which is unknown). The PIs perform the best (RMSE 0.27 log units, CCP 93% internal and 0.43 log units, CCP 90% external), followed by the NNRTIs (RMSE 0.45 log units, and CCP 93% internal and 0.49 log units, CCP 91% external) and then the NRTIs (RMSE 0.31 log units, CCP 80% internal and 0.52 log units, CCP 68% external). The range of Log FC values present in the dataset is the largest for the NNRTIs, followed by the PIs and then the NRTIs.

The second step was the generation of models on 70% of the dataset as the learning curves showed this to be the optimal split size to get a reliable performance estimate for these models. While these 70% models give an estimate of the ability of the models to perform future predictions successfully, other additional forms of validation should also be included as we will show later on. [36] The RMSE for sequences that were present in the training set, however not in combination with the same drug, was 0.27 (PIs, Figure 2C), 0.31 (NRTIs, Figure 2B) and 0.45 (NNRTIs, Figure 2A), with an R_0_^2^ 0.89 (PIs, Figure 2C), 0.79 (NNRTIs, Figure 2A), and 0.75 (NRTIs, Figure 2B). Hence, we found that PCM was overall able to extrapolate the Log FC values for individual pairs of drug and mutant not encountered in the training set with a reliability that slightly better than the assay reliability of the current dataset (approximately 0.5 log units). Hence, PCM is on this dataset able to extrapolate to novel drug-mutant pairs when the drug and mutant in question are only present in the training set individually, and not in the combination, as shown in the test set (internal validation).

For sequences not present in the training set (representing predictions for previously unseen patients, or genotypes) the RMSE obtained by the model was 0.43 (PIs, Figure 2F), 0.49 (NNRTIs, Figure 2D) and 0.52 (NRTIs, Figure 2E) with an R_0_^2^ of 0.74 (NNRTIs, Figure 2D), 0.71 (PIs, Figure 2F) and 0.33 (NRTIs, Figure 2E), respectively. Hence, PCM is on the current dataset also able to extrapolate the Log FC values for individual pairs of drug and mutant not encountered in the training set with reliability comparable to assay reliability when the mutant in question is not present in the training set (External validation, for validation plots per individual drug please see Figures S2, S3, S4).

### PCM model validation (improvement over alternative approaches on this set)

We also pursued two alternative approaches to model the current dataset. These steps provide an estimate of the added value of PCM itself rather than size of the dataset. Our first validation was calculating the average of the Log FC values of mutants for which we had multiple drug Log FC measurements. Subsequently this average Log FC value was used as a predictor for the drugs that were left out. This method was called Log FC scaling and is similar to a benchmark we used in previous work. [33] By comparing the average Log FC value to the value measured on the sequence left out, the chemical descriptor component of PCM is removed. Moreover the main contributors to changes in Log FC in this method are those causing cross resistance as the effects on individual Log FC values are ignored. We aimed to leave out 30% when multiple measurements were possible, when only three or two measurements were available we left out one drug measurement. The results are included as Supporting table S2, where PCM on average has a 50% lower RMSE (on average 0.38 log units for PCM versus 0.56 log units for scaling).

Likewise we trained individual models per drug using only the sequence descriptors, hence this approach is conceptually identical to Virtual Phenotype or Geno2Pheno models (shown as ‘sequence only’ in supporting Table S2). The goal here was to ensure that including chemical (ligand) information indeed improves model performance. Indeed, we found that also here PCM outperforms sequence only models in all drug classes. In all cases the prediction error improves by approximately 11% (with a similar improvement of correlation coefficient). This improvement is significant for the NRTIs when performing a paired t-test (RMSE, P<0.01; R_0_^2^, P<0.01) and PIs (RMSE, P<0.01; R_0_^2^, P<0.05). The difference was not significant for the NNRTIs, while PCM did outperform sequence only models (RMSE, P = 0.33; R_0_^2^, P = 0.14). We think this is mainly due to the large chemical diversity of the NNRTI drug class, which are similar in pharmacophoric properties but display a diverse collection of scaffolds. Since we use two dimensional chemical descriptors rather than three dimensional, PCM cannot reach the large performance difference shown for PI and NRTI. This is supported by the fact that the chemically most different NNRTI, ETR, is the only one that has a lower performance in PCM models (similarity on average 0.36, Supporting Table S7). Yet, the combination of the bioactivity space for individual NNRTIs is successful as NNRTIs are known to be sensitive to cross resistance, this is captured by PCM.

### PCM model validation (Clinical Cut-offs)

In order to investigate clinical relevance of our work, we next incorporated the actual clinical cut-off (CCO) values. These values describe the expected response of a patient to treatment with a certain drug based on the HIV genotype (the used clinical CCO values are given in supporting Table S10 and S11). When we apply the CCOs to our model predictions, our models achieve an overall correctly classified percentage (CCP) of 95% for the inhibition of mutant sequences present by a drug not present for that sequence in the dataset (Figure 2).

For the sequences not present in the training set, 91% was predicted correctly (supporting Table S3 and S4). More specifically per class, the PI scored the best (93% correct for internal validation and 90% correct for external validation), followed by NNRTIs (93% correct for internal validation and 91% correct for external validation), and lastly the NRTIs (80% correct for internal validation and 68% for external validation). However, it should be noted that for the NRTIs only a small number of sequences was available as validation, and all were not very resistant, possibly leading to a biased validation.

We can conclude that even prediction on sequences not present in the training set was possible, albeit slightly less than the internal validation (RMSE 0.34 log units when the sequence is known versus 0.48 when it is not). To further find the limitations of this extrapolation we employed leave-one-sequence-out (LOSO) validation.

### Leave-One-Sequence-Out validation (LOSO)

**Figure 3:**
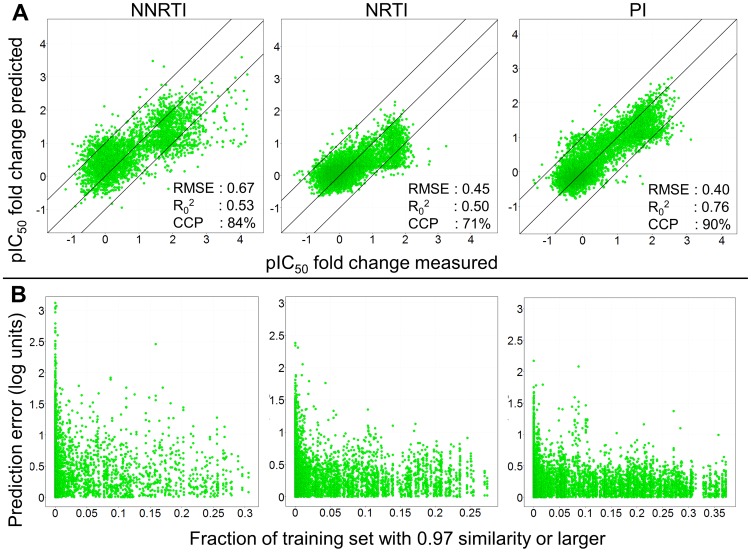
The model performance in the LOSO experiments. (A) The figure visualizes the measured Log FC for a mutant – drug pair on the x-axis. The y-axis shows the Log FC predicted for that mutant – drug pair by a model that was trained without that particular pair. Again the PIs perform the best (RMSE 0.40 log units, R_0_^2^ 0.76, and CCP 90%) followed by the NNRTIs (RMSE 0.67 log units, R_0_^2^ 0.53 and CCP 84%) and then the NRTIs (RMSE 0.45 log units, R_0_^2^ 0.50 and CCP 74%). (B) The density to the training set as a measure of applicability domain provides a useful estimate to predict model reliability. The x-axis shows fraction of the training set that has a similarity of 0.97 or higher to a specific mutant – drug pair. If this fraction is larger, then the prediction error (y-axis) for that pair becomes smaller as the model is better able to extrapolate from the training set. Since this fraction can be calculated before any model prediction is made, a maximally allowed prediction error can be predetermined before any model predictions are made.

LOSO validation is unique to proteochemometric approaches, since it enables the prediction of compound activities for *entirely novel genotypes* (or patients), hence estimating which treatment would be most likely to succeed in a given treatment situation. For computational reasons, our approach used a subset of approximately 1,000 mutants from the full set (4% (PR) and 9% (RT) of the total dataset, respectively). Each of these sequences was left out, and a model was trained on the remaining sequences; results are shown in Figure 3. Again, the PCM technique overall provides rather robust in modeling the current dataset. Best performance can be observed for the PI model (with an RMSE of 0.40 log units, R_0_^2^ of 0.76 and CCP 90%), followed by the NNRTIs (RMSE of 0.67 log units, R_0_^2^ of 0.53 and CCP 84%) and the NRTIs (RMSE of 0.45 log units, R_0_^2^ of 0.50 and CCP 71%). The finding that PIs and NRTIs are easier to model than NNRTIs is in line with our finding above. What should be noted is that the NNRTI model tends to slightly underpredict the Log FC values that have been measured with a Log FC above 3.0. While those values are correctly predicted to be above 1.0 (which is an important prediction to have by itself in practice), the numerical correlation between predicted and experimental values leads to a slight, but consistent under prediction of activities in this value range.

Crucial for the application of computational models is an estimate in which cases the model can be trusted, and where it is likely to fail. In this spirit, the ‘Applicability Domain’ of computational models has become an important topic recently;[37] however, so far it was mainly applied to the chemical domain. This concept was extended in the current work, given the nature of PCM models, also to the protein target or biological domain where special considerations need to be taken into account. Since we are dealing with a large set of viral mutants we are unable to define a single similarity to a WT to get an idea of the applicability domain. Therefore, we chose to define the applicability domain based not only on the distance to the training set, but also on the density of neighbors in the training set (See Methods section for details). At a similarity threshold of 97% each sequence is hence assigned a density score between 0 and 1 (0 corresponding to no sequences with a similarity of at least 97%, and 1 corresponding to all sequences in the dataset having more than 97% similarity to the sequence under consideration).

Figure 3 visualizes the ‘Neighborhood Behavior’;[38] if the fraction of sequences having this similarity of 97% (X-axis) is larger (closer to 1), the maximal encountered prediction error (RMSE, y-axis) is lower (closer to 0 log units). This means that if the model can extrapolate from a larger number of sequences having a similarity of 97% or higher, the predictions become more reliable. Performance of a practically useful model would require the largest error to be below 1 log unit; hence, given this requirement, the density of sequences in the training set should be larger than 0.15 (for PIs and NRTIs) and larger than 0.25 (for NNRTIs), respectively. Due to this numerical quantification of the ‘Applicability Domain’ of the model, in *biological space*, we are now able to judge in which situations the model *will be* applicable (*i.e.* is likely to generate reliable results), and in which situations it *is not* which is of crucial importance in order to gain trust into computational models.

### LOSO validation (Clinical Cut-offs)

Further exploring the clinical relevance of this work, the CCO's were again applied to model predictions also in the case of the LOSO experiments. Overall the model reached a CCP of 81% of the individual mutant – drug pairs. Moreover, 12% of the total predictions were overpredicted, and only 7% underpredicted. Hence our models perform robust also on sequences *that are entirely novel to the model* (supporting Table S5). For the individual classes, the image is very similar to that in the external validation, the PIs perform the best (90% correct), followed by the NNRTIs (84% correct) and lastly the NRTIs (71% correct).

In the text above we have thoroughly validated our models and they have shown to be robust in modeling HIV resistance to PIs, NNRTIs and NRTIs. This was confirmed for known sequences in an unknown combination with a drug but also for unknown sequences in an unknown combination with a drug. Hence we conclude that our models describe the drug – target interaction space, therefore it is very interesting to investigate how our models actually derive these Log FC values from the contributions individual mutations make.

### PCM compared to sequence only linear models

To compare the performance of our PCM models with state of the art models trained on sequence data only and to place the results of our work in perspective, we used a dataset previously published by Van der Borght *et al.* [39] We explicitly selected for each class the 150 sequences that were predicted most inaccurate, representing the most difficult sequences to predict (these were mutants that seem to exhibit a different resistance profile). Moreover, most of these sequences contained mixtures (several mutations present on a single position) that had been discarded from our PCM training set. The purpose of this validation was therefore twofold, to assess the performance of PCM when compared to sequence only models, and secondly to assess if the PCM models can deconvolute the effect of individual mutations to make accurate predictions for mixture sequences.

### PCM model performance compared to previously published models

**Figure 4:**
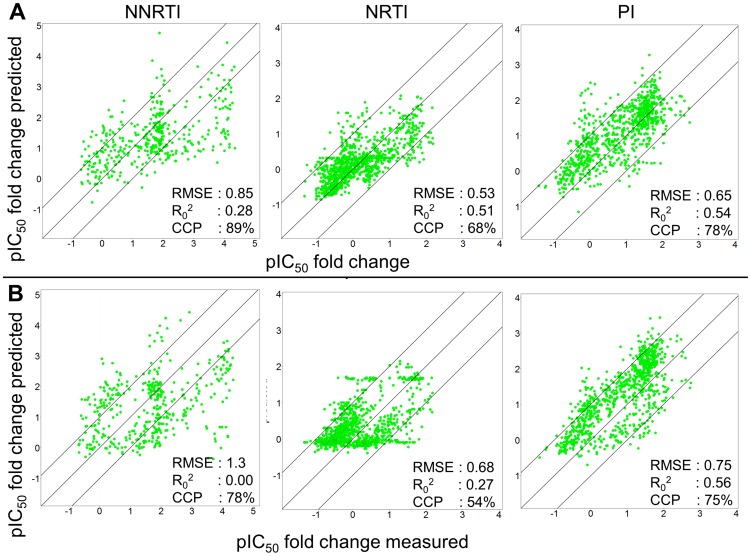
Performance of PCM based models compared with sequence based models for the 150 most difficult sequences as published by Van der Borght et al. The PCM models (A) perform better as they have a lower prediction error for each drug class (0.53 log units versus 0.68 log units for the NRTIs; 0.65 log units versus 0.75 log units for the PIs, and 0.85 log units versus 1.3 log units for the NNRTIs) than the sequence based models (B). Clearly the NNRTIs are most difficult to predict. Note that these sequences contain a large fraction of mixture sequences, which were not present in the PCM training set but were present in the sequence only training set. In addition, the PCM models also reach a higher CCP compared to the sequence only models.

The results of this validation are shown in Figure 4, Table 1 and Supporting Table S2. Our PCM models clearly outperform sequence only models. For each class the PCM models predict the Log FC more accurately. This is indicated by the smaller RMSE (0.53 log units versus 0.68 log units for the NRTIs; 0.65 log units versus 0.75 log units for the PIs, and 0.85 log units versus 1.3 log units for the NNRTIs) and also by a higher CCP (68% versus 54% for the NRTIs, 78% versus 75% for the PIs, and 89% versus 78% for the NNRTIs). For several PIs, the sequence only models perform marginally better when measuring by the correlation coefficient; however as these values are systematically slightly overpredicted in the sequence only models, PCM still performs better.

**Table 1:** Performance of PCM compared to sequence only models published by Van der Borght *et al.*

RMSE (Log units)	R_0_^2^	RMSE Sequence only (Log Units)	R_0_^2^ Sequence only	Grouping
0.66 (±25)	0.41 (±0.19)	0.80 (±0.29)	0.22 (±0.41)	Drug (average)
*0.65*	*0.54*	*0.75*	*0.56*	*PI (Class)*
0.59	0.64	0.67	0.66	APV
0.67	0.57	0.83	0.50	ATV
0.80	0.39	0.79	0.49	DRV
0.62	0.54	0.76	0.59	IDV
0.65	0.60	0.83	0.67	LPV
0.63	0.49	0.73	0.48	NFV
0.63	0.52	0.76	0.57	SQV
0.53	0.41	0.55	0.42	TPV
*0.85*	*0.28*	*1.3*	*0.00*	*NNRTI (Class)*
0.93	0.39	1.1	0.10	ETR
1.5	0.12	1.8	0.00	EFV
0.72	0.00	0.95	0.00	NVP
*0.53*	*0.51*	*0.68*	*0.27*	*NRTI (Class)*
0.67	0.49	0.83	0.31	3TC
0.41	0.46	0.53	0.15	ABC
0.59	0.45	0.75	0.20	AZT
0.45	0.27	0.54	0.00	D4T
0.42	0.35	0.51	0.10	DDI
0.65	0.51	0.90	0.20	FTC
0.43	0.36	0.59	0.00	TDF
***0.66***	***0.42***	***0.80***	***0.30***	***Overall***

Validation parameters were calculated using different forms of grouping to give an unbiased error estimate. The table shows that our PCM models perform better than sequence only models. This is indicated by the regression validation parameters RMSE and R_0_^2^. While it should be noted that for some of the PIs, the sequence only models tend to have a slightly higher R_0_^2^, they also have a much higher RMSE.

Furthermore, when we limit ourselves to only predicting the Log FC for mutant mixtures, PCM still outperforms sequence only models (supporting Table S2, supporting Table S6 and supporting Figure S5). This is even the case while our PCM models were trained without mixture sequences in the training set whereas these were present in the training for the sequence only models. A large fraction of these mixtures sequences show a low value for the 97% similarity density, hence we would expect the models to perform suboptimal on these sequences. The applicability domain measure therefore also holds in this case. These results underline the added value of PCM models over sequence only models and hence we also wanted to interpret these models.

### Model interpretation (known resistance mutations)

The aim of this feature importance investigation was to explain the *average* reduction in drug affinity that the presence of an individual mutation causes. Firstly, we investigated the effect of several known mutations from literature. To this end we compared the features selected as being significant by our model to the mutational overviews published by Johnson *et.al.* [6], [40]

**Figure 5:**
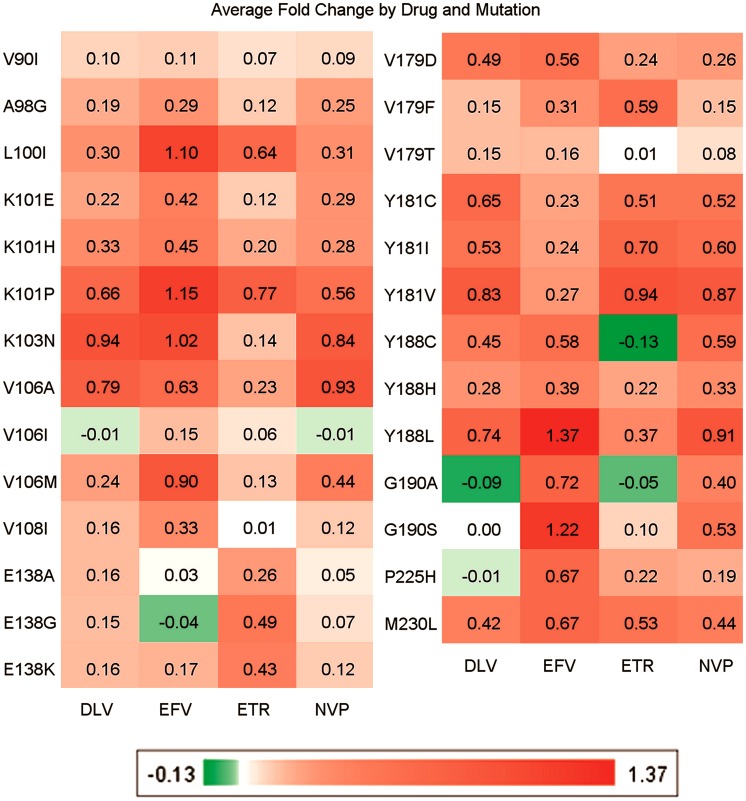
Model interpretation, known mutations that lead to NNRTI (cross) resistance. The pattern produced by our model correlates with literature. [6], [18] In particular the specific profiles of V106I, Y181C and G190A are reproduced well. Values in the cells represent Log FC.

Figure 5 shows the impact of selected mutations on NNRTI affinity. Overall, while there is a significant amount of cross-resistance, each of the NNRTIs still possesses its own distinct resistance profile, in agreement with the importance of personalized HIV treatment approaches. Furthermore, the impact of individual mutations varies per drug and is in line with literature data. [6], [18] Red indicates that the presence of this mutation leads to a higher Log FC on average, whereas green indicated that the presence of this mutations leads to a lower Log FC on average, and white indicates that this mutation has little effect on the Log FC (For an explanation of the abbreviations see supporting Table S1). For instance, mutation K103N has a rather specific pattern as it confers resistance to Nevirapine, Efavirenz, and Delavirdine but not to Etravirine. [6], [18] This pattern is reproduced by our model. Furthermore, V179F is known to lead to Etravirine resistance but to have less effect on Nevirapine, Efavirenz, and Delavirdine, [6], [18] a resistance profile that can also be reproduced based on our dataset. Some mutations are slightly underestimated, these include V90I and V106I. Another interesting observation is that mutations Y188C and G190A are predicted to render HIV *more sensitive* to Etravirine according to our model. This finding is in agreement with work by Vingerhoets *et al.* [41]

Related analyses for NRTI resistance and PI resistance have been included in the supporting information (supporting Figure S6 and supporting Figure S7). Specific NRTI mutations that were accurately reproduced include K65R, Q151M, and T215Y, while mutations M41L and M184V are slightly underestimated, compared to previous studies. [6] For the PIs mutations that are accurately reproduced include D30N, I50L, V82S, and I84, while the I64L and I93M mutations are assigned less importance than in previous work. [6]

Hence, the PCM models applied in this study are able to reproduce known resistance patterns as outlined above. This led us to the next step of the study, the identification of *novel* mutations (present in our dataset but not previously published) which are found to confer cross resistance to antiretroviral treatments. This work is similar to previous work by Van der Borght *et al.* [39] but here we focus on both cross resistance conferring mutations *and* drug specific mutations. Furthermore we apply the method to all three major classes of anti-HIV drugs rather than one and can do so directly from our models.

### Model interpretation (cross resistance-conferring mutations)

To identify cross-resistance as part of the current study, we were limiting ourselves to mutations that have a *negative* effect on the majority of drugs in a single class. However, in case of particular interest in the resistance profile of a particular drug this analysis can also be performed on the individual-drug level subsequently.

We selected mutants based on the following conditions: occurrence in the dataset more than once; average Log FC for all compounds above 0.4; standard deviation over this average below 0.4. Known mutations as published in literature were discarded. [6], [40], [42], [43] With these filters a number of novel resistance conferring mutations could successfully be identified which are listed in Table 2–4 (For an explanation of the abbreviations see supporting Table S1). Mutations identified have a high impact on drug affinity and which lend themselves to experimental validation, for instance in the case of NNRTI and NRTI resistance conferring mutation T216M. The full set of individual mutations (both known and novel) and their effect is included in the supporting Information as delimited text files (Dataset S1).

**Table 2:** Novel resistance conferring mutations derived from the dataset (NNRTI).

Mutation	DLV	EFV	ETR	NVP	Average Log FC
P9T	0.36	1.01	0.65	0.46	0.62
E79D	0.34	0.55	0.61	0.34	0.46
K101S	0.38	0.73	0.31	0.44	0.47
K102Y	0.72	0.53	0.47	0.77	0.62
S156A	0.8	1.2	0.76	0.67	0.86
M164L	0.26	0.89	0.51	0.62	0.57
T216M	0.97	1.47	0.01	0.84	0.82
Y232H	0.47	0.62	0.36	0.47	0.48
R307M	0.92	0.14	0.28	0.35	0.42
Average Susceptibility	0.58	0.79	0.44	0.55	-

The value in the different drug columns indicates the average Log FC in the presence of this mutation. While these mutations have been selected to confer some resistance to all NNRTIs, each drug still has a distinct profile. Efavirenz is the most sensitive (average Log FC 0.79) and Etravirine the least (average Log FC 0.44) with Nevirapine (average Log FC 0.55) and Delavirdine (average Log FC 0.58) in between.

**Table 3:** Novel resistance conferring mutations derived from the dataset (NRTI).

Mutation	3TC	ABC	AZT	D4T	DDC	DDI	TDF	FTC	Average Log FC
I63V*	0.22	**n/a**	1.07	0.53	**n/a**	0.52	0.01	0.36	0.45
I202M*	0.23	**n/a**	0.73	0.68	0.51	0.57	0.45	0.39	0.51
R206M	0.90	0.42	0.54	0.01	0.15	0.17	0.24	0.92	0.42
T216M	0.88	0.51	0.66	0.12	0.20	0.27	0.38	0.94	0.50
E298K*	0.33	0.43	0.44	0.38	0.65	0.32	**n/a**	**n/a**	0.43
Average Susceptibility	0.51	0.45	0.69	0.34	0.38	0.37	0.27	0.65	-

The value in the different drug columns indicates the average Log FC in the presence of this mutation, when not available in the dataset the value is denoted ‘n/a’. Mutations indicated with an asterisk were incompletely tested on all drugs in the dataset. Like the NNRTI resistance mutations, each mutation displays a different resistance profile over all drugs. AZT is seen to be the most susceptible (average Log FC 0.69) and TDF the least susceptible (average Log FC 0.27).

**Table 4:** Novel resistance conferring mutations derived from the dataset (PI).

Mutation	APV	ATV	DRV	IDV	LPV	NFV	RTV	SQV	TPV	Average Log FC
Q18N	0.55	0.52	0.56	0.61	0.58	0.49	0.56	0.50	0.65	0.56
V32T*	0.63	0.65	0.07	0.67	0.45	0.67	0.68	0.81	**n/a**	0.58
N88G	0.39	0.97	-0.27	0.77	0.18	1.14	0.10	0.49	0.22	0.44
Average Susceptibility	0.52	0.71	0.12	0.68	0.40	0.77	0.45	0.60	0.44	-

The value in the different drug columns indicates the average Log FC in the presence of this mutation, when not available in the dataset the value is denoted ‘n/a’. Mutations indicated with an asterisk were incompletely tested on all drugs in the dataset. Here Nelfinavir is the most susceptible (average Log FC 0.77) and Darunavir the least (average Log FC 0.12).

### Model interpretation (drug-specific resistance-conferring mutations)

**Figure 6:**
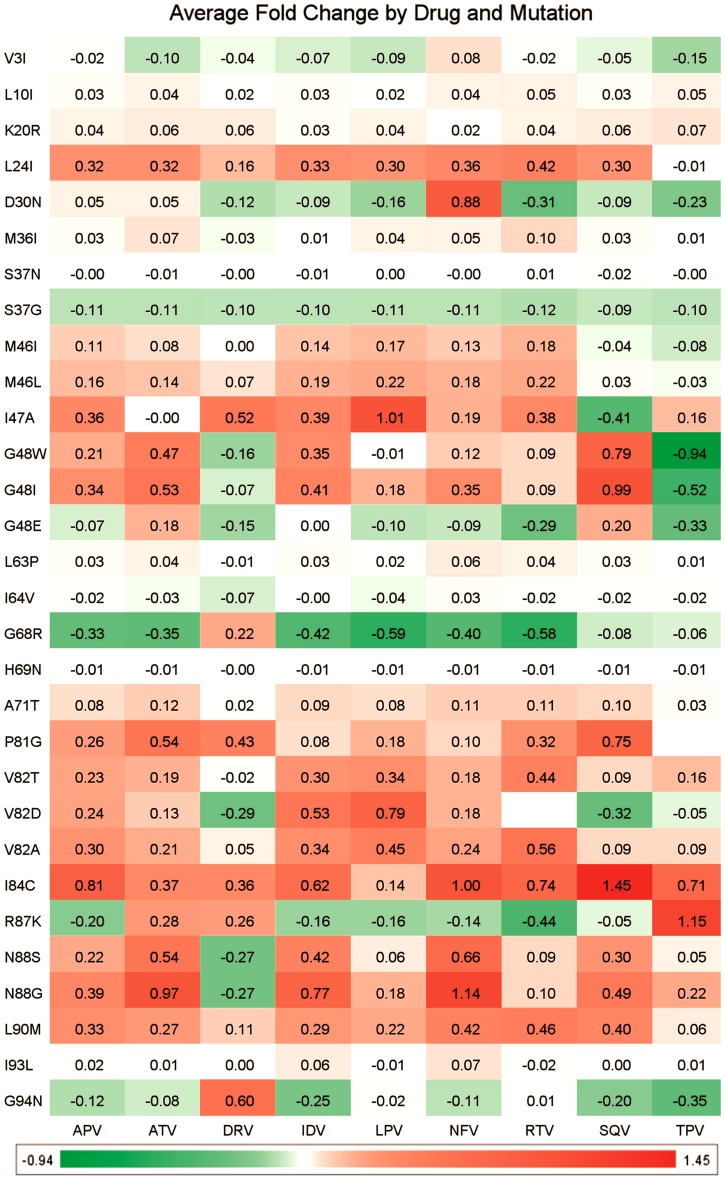
Model interpretation, mutations leading to PI specific resistance. Shown are the 30 mutations that have the most diverse effect over the different members of the PI drug class. The figure contains a number of known mutations (e.g. M46L, [6] A71T, [6] V82A, [6] V82S [6]) but also several novel mutations (e.g. G48W, N88G). Values in the cells represent Log FC.

We furthermore analyzed not only mutations that cause cross-resistance, but also those with a particular effect on a specific drug treatment alone. The goal here was to identify mutations that lead to large resistance for one drug but are still sensitive for another drug from the same class. Hence this knowledge can be of high importance in a clinical setting. For the PIs the 30 most interesting mutations (defined as those mutations that have the most diverse effect on the different drugs), are shown in Figure 6 (while corresponding figures for the NNRTIs and NRTIs are included in supporting Figure S8 and supporting Figure S9). In those figures we can observe several mutations that lead to resistance for a single drug (Log FC on average >0.5) and at the same time lead to higher sensitivity for another drug (Log FC on average <0.0).

For instance, the G48W mutant is sensitive to Darunavir and Tipranavir, while showing some degree of resistance to all other PIs. Furthermore, R87K is resistant to Atazanavir, Darunavir, and Tipranavir, but sensitive to all other drugs in the dataset. This could indicate that at this point the mutant has over-adapted to the host environment, including the drug, hence rendering the mutant very sensitive to changes in this environment. Finally, N88G seems to only be sensitive to Darunavir, while conferring resistance to all other PIs in the dataset. Information of this type is of high relevance to prescribe the optimal drug for an individual patient, by being able to link the viral genotype to the clinical phenotype in a real-world situation. Applying these models in a real world situation on unseen clinical data is exactly what we implemented in the following paragraphs.

### Validation on unseen data (Stanford University data)

Given a sequence of PR and RT (and hence, a viral genotype of a patient to be treated), our models are able to predict which drugs will be least influenced by resistance, as measured via the lowest Log FC. To accurately estimate our model performance in prospective predictions, in the final step of this study we performed a validation on unseen data. Apart from only focusing on unseen data, in order to establish agreement of our modeling procedure with other approaches, we also employed data from an entirely different source – namely, for sequences obtained from the Stanford University HIV Drug Resistance Database (Stanford Set). [18], [44] The set we used has also been included as supporting information (Dataset S2).

### Validation on unseen data (model performance)

**Figure 7:**
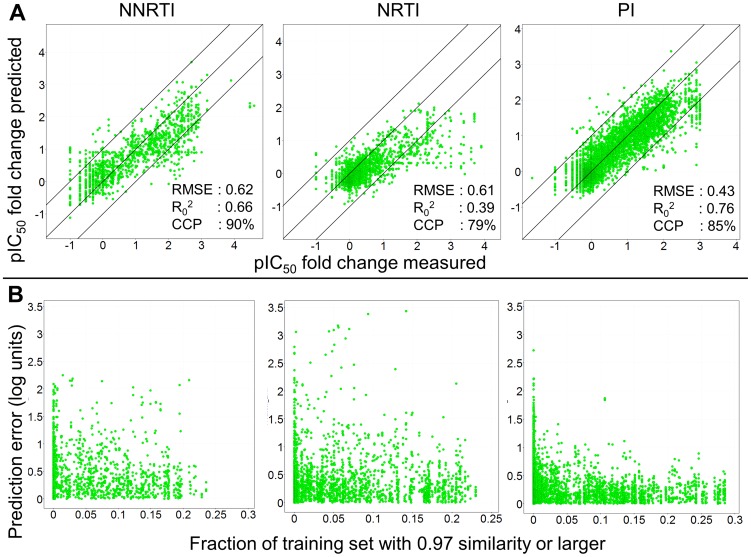
Model performance predicting the Stanford University dataset. (A) The isolates predicted were not included in the training set, still performance is robust. Based on CCP, the NNRTIs perform the best (RMSE 0.62 log units and CCP 90%), followed by the PIs (RMSE 0.43 log units and CCP 85%) and then the NRTIs (RMSE 0.61 and CCP 79%). (B) The density to the training set as a measure of applicability domain provides a useful estimate to predict model reliability. The x-axis shows fraction of the training set that has a similarity of 0.97 or higher to a specific mutant – drug pair. The larger this fraction, the smaller the prediction error (y-axis) for that pair as the model is better able to extrapolate from the training set.

Applied to the Stanford Set, the PCM models developed in the current work show an average RMSE of 0.52 log units, with the average R_0_^2^ being 0.59. Compared to the models above, this is a slightly larger error compared to the validation on Virco data, which was below 0.50 log units. (It should be noted that this is very diverse data, including historical literature data of which we cannot estimate reliability.) The PI model again performs the best (with an RMSE of 0.43 log units and an R_0_^2^ of 0.76), while the NNRTIs are predicted with the largest error (with an RMSE of 0.62 log units and an R_0_^2^ of 0.66), which is the result of a number of outliers (see Figure 7 and explanation below). The NRTI model exhibits the lowest correlation coefficient (R_0_^2^ 0.39 and RMSE 0.61 log units), mostly due to the relatively small range of Log FCs present in the dataset. However, also in this case we observe a correlation between the density of sequences with a 97% similarity in the training set and modeling error, also allowing us to establish the Applicability Domain of the model throughout.

### Validation on unseen data (discussion of outliers)

With the NRTIs and some NNRTIs there are outliers to the Applicability Domain we established, meaning that expected and observed errors exhibited some differences. (Note that this is usually the case, the Applicability Domain concept being a concept based on error distributions and likelihoods, not certainties, that a given error will be obtained in a given situation.) It was found that these outliers were obtained from only a small number of references (RefIDs) from the Stanford DB. References 369, 414, 649 all contained the M184V and T215Y mutations that are also known to differ between AVG and Phenosense. Furthermore there was a major discrepancy between the Log FC values reported for AZT on similar mutant which was >2000 (log value 3.3) in one reference, while being reported as low as 28 (log value 1.4) from another source. [45], [46], [47] Reference 789 contained a sequence carrying a deletion at position 69, which was not taken into account by our model. [48] Reference 947 linked to unpublished data and could therefore not be verified. Finally, reference 1261 is underpredicted for both the NRTI tested sequences and NNRTI tested sequences and we could not identify an apparent cause for this behaviour. [49] (More detailed results are listed in Table 5.) The table shows that performance per drug is very good with a low RMSE (an average RMSE of 0.54 log units; with two outliers, AZT and FTC, exhibiting an RMSE of >0.90 log units). Overall, when the results are grouped per literature reference number (which is included in the dataset) the average quality decreases and the standard deviation increases, indicating that differences between reported Log FC changes in literature exist and this could adversely affect model performance.

**Table 5:** Performance in validation on isolates not present in the original dataset.

RMSE (Log units)	R_0_^2^	Correctly Classified Percentage	Overpredicted Percentage	Underpredicted Percentage	Grouping
0.54 (±0.28)	0.56 (±0.27)	0.85 (±0.13)	0.09 (±0.11)	0.06 (±0.09)	RefID (average)
0.45 (±0.33)	0.62 (±0.34)	0.84 (±0.24)	0.11 (±0.20)	0.06 (±0.15)	IsolateName (average)
0.44 (±0.34)	0.62 (±0.34)	0.84 (±0.24)	0.10 (±0.20)	0.06 (±0.15)	SeqID (average)
0.54 (±0.18)	0.58 (±0.19)	0.83 (±0.10)	0.11 (±0.10)	0.06 (±0.06)	Drug (average)
*0.44*	*0.75*	*0.85*	*0.11*	*0.03*	*PI (Class)*
0.43	0.74	0.86	0.10	0.04	ATV
0.37	0.75	0.72	0.28	0.00	IDV
0.39	0.83	0.91	0.03	0.06	LPV
0.44	0.76	0.9	0.05	0.04	NFV
0.44	0.78	**0.91**	**0.05**	**0.03**	**RTV**
0.49	0.75	**0.88**	**0.07**	**0.05**	**SQV**
0.52	0.38	0.70	0.20	0.02	TPV
*0.68*	*0.65*	*0.89*	*0.05*	*0.06*	*NNRTI (Class)*
0.64	0.63	**0.83**	**0.10**	**0.07**	**DLV**
0.60	0.70	1.00	0.00	0.00	EFV
0.76	0.65	0.87	0.04	0.09	NVP
*0.61*	*0.39*	*0.79*	*0.12*	*0.09*	*NRTI (Class)*
0.47	0.49	0.85	0.09	0.07	ABC
0.90	0.56	0.84	0.09	0.07	AZT
0.41	0.37	0.64	0.12	0.23	D4T
0.42	0.35	**1.00**	**0.00**	**0.00**	**DDC**
0.39	0.41	0.74	0.17	0.10	DDI
1.01	0.66	0.85	0.00	0.15	FTC
0.44	0.12	0.66	0.30	0.04	TDF
***0.53***	***0.65***	***0.84***	***0.10***	***0.06***	***Overall***

Validation parameters were calculated using different forms of grouping to give an unbiased error estimate. Class wide values are indicated in italic and the global average performance is indicated in bold and italic. For larger groups (RefID, SeqID, Isolatename and per drug) the average value and standard deviation are given. For three drugs (RTV, DLV, DDC) no Virco cut-off was available, here the Stanford cut off was used for both, for SQV no Stanford cut-off was available so the Virco cut-off was used for both. The table shows that our PCM models perform robustly in predicting the Log FC as indicated by the regression validation parameters RMSE and R_0_^2^. More importantly, the correctly classified percentage is 84% overall.

### Validation on unseen data (Clinical Cut-offs)

As each assay uses its own set of CCO values tuned for the respective assay we used values supplied by Virco and Rhee *et al.* for the Virco set and the Stanford set respectively. [44] Our model classifies the response correctly in 84% of the cases (Table 5). The average performance when grouped per individual drug class was very good (PI 85%, NNRTI 89% and NRTI 79%). Also noteworthy is that the model bias is towards over prediction rather than under prediction, something that is not always mentioned in literature but is especially relevant in a clinical setting.

Previous work on a *high quality filtered subset* of our Stanford DB set reached 80% correct predictions of phenotype from genotype on average (PI 78%, NNRTI 83% and NRTI 75%). [44] Other work indicates that an expert panel reaches up to 44% correct predictions. [50] The two outliers in the NRTI class are d4T and TDF, for which an apparent discrepancy between AVG data and Phenosense data has previously been described. [51]

### Conclusions

In this work we report the construction of robust PCM models, based on 300,000 bioactivity data points measured against different HIV genotypes. In total, the model contained information on a total of 4 (NNRTI), 8 (NRTI) or 9 (PI) drugs combined with 10,700 (NNRTI) 10,500 (NRTI) or 27,000 (PI) mutants. Given the nature of the PCM modeling procedure employed in this work, we were able to combine all resistance profiles of the three above drug classes in three single models, hence focusing on very large *target space* (tens of thousands of different proteins) in this work. Both in internal validation and validation on unseen data our model showed performance comparable to assay reliability and better than sequence only models; moreover, model interpretation has been performed to identify *novel* resistance-conferring mutations that lead to resistance to *all* drugs in a class, such as T216M in the case of RT. In addition, we can use these models to find mutations that lead specific sensitivity (G48W in PR) or resistance (G68R in PR) to a single drug within a class.

Another application of our models is the support of personalized drug regimen predictions. We have shown that our models are able to predict clinical resistance with a high degree of reliability. This reliability is formed by a 95% CCP when predicting clinical response for Antivirogram data, which is the assay models were trained on, similar studies reached 80% CCP when predicting values for the assays they trained on. Furthermore, the CCP and is as high as 81% when predicting clinical response for *unknown* mutants. The novelty is formed by reliable predictions on *unknown* mutants and even *unknown mixtures*. Finally, the CCP is 84% when predicting clinical response for clinical isolates obtained from very diverse sources (including historical literature data and data from different assays), indicating that the model is robust and predictive.

We attribute the better performance of PCM to two reasons. Firstly our models are trained on a very large co-linked dataset. This large training set not only minimizes the influence and bias caused by single experimental error, it also allows the model to detect global patterns that are consistent over both genotype (sequence similarity) and chemo type (drug similarity). The second reason is related to the first, as the encoding of the *full sequences* using physicochemical properties rather than presence or absence of mutations allows for a better similarity measure between two sequences.

## Methods

### Dataset

The main dataset was obtained from Virco (Beerse, Belgium) and consisted of mutants (both PR and RT sequences) and fold change (Log FC) in pIC_50_ (log units) data in the AVG assay collected by Virco up to January 2011. [12], [25], [28] Mixtures, consisting of multiple mutants that were identified in a single clinical isolate) were removed from the set and the total size of the dataset is listed in Table 6. The Log FC data was used as is, since it already consisted of log units difference to a single mutant defined as wild type. The wild type was defined as the HXB2 isolate (Uniprot accession P04585 and Genbank accession K03455), shown in Table 6 are the mean number of mutants present per sequence compared to HXB2. [22]

**Table 6:** Description of the dataset used in the current study (Obtained from Virco).

Target	Amino acids	Binding Site	Drug Class	Drugs	Mutant Sequences	Data points	Mean number of mutations
Reverse Transcriptase	400*	Orthosteric	NRTI	8	10,501	72,727	22 (±9)
Reverse Transcriptase	400*	Allosteric	NNRTI	4	10,723	35,249	22 (±9)
Protease	99	Orthosteric	PI	9	27,081	180,162	10 (±6)

* For Reverse Transcriptase only the first 400 amino acids were sequenced. The total size of the dataset is unlike any other dataset used in PCM. The number of mutations shown in the last column is the average per sequence and standard deviation when compared to HXB2.

Furthermore, little duplicate sequences were actually in the dataset, specifically: NNRTI 1,501 duplicates (of 10,723 sequences), NRTI 1,411 duplicates (of 10,501 sequences), PI 9,803 duplicates (of 27,081 sequences).

### Mutant descriptors

Sequences were subsequently encoded using the first three Z-scales. [52], [53] The Z-scales are a previously published set of descriptors that characterize the physicochemical properties of the amino acids side chains. The resulting scales correlate to lipophilicity, size and polarity for each amino acid. For PR the full sequence was used and for RT only the first 400 amino acids were sequenced as the final 160 residues form an RnaseH domain and are not directly relevant in (N)NRTI resistance. These Z-scales were subsequently used to train models.

### Drug descriptors

Structures of the drugs were normalized and ionized at pH 7.4, they were assigned 2D coordinates and subsequently converted to Scitegic circular fingerprints of type ECFP_8, ECFP_10 and ECFP_12 (depending on drug class, as described below). [54], [55] All this was done in Pipeline Pilot Student Edition version 6.1.5. [56] Circular fingerprints employ all possible substructures for a molecule up to a predefined maximal bond diameter. Each substructure is then encoded as a bit given the value ‘1’ when present and ‘0’ when absent. The reason for employing circular fingerprints is that they have previously been shown to give very high retrieval rates in comparative studies. [57] The NRTI dataset employed ECFP_10 fingerprints while the NNRTI dataset used ECFP_8 and the PI dataset ECFP_12 fingerprints.

In order to create a numeric descriptor for each drug, a similarity matrix was constructed using the fingerprints and based upon the Tversky Similarity coefficient. [58] Here fingerprints were converted to a fixed length array of counts with maximal length of 256 bits where the most descriptive bits were sorted to be at the beginning of the array. The value for α was 0.1 and the value for β was 0.9, putting more weight on the unique features of the target molecules compared to the reference molecule. For each drug in a class, the similarities to all other drugs from that class were then used as a descriptor (Tables S7, S8, S9).

### Machine learning

Models were constructed in the academic version of Pipeline Pilot 6.1.5 using the R-statistics package. [59] Support vector machines (SVM) with a radial basis function kernel as coded in the e1071 package were used for model creation. [60] Parameters gamma and cost were tuned over an exponential range and epsilon was set at 0.25. It has been shown that setting epsilon to the approximate data error is the optimal value for training. [61] The optimal model was determined using 5 fold cross validation before proceeding to external validation of the model. The parameters used for validation were R_0_^2^, R^2^, and RMSE. [36], [62]

### Density based applicability domain

As our models are trained on a database of different HIV mutants, applicability domains based on a single wild type sequence are expected to perform sub optimal. Rather we choose to determine the applicability domain based on the density of the nearest neighbors in the training set. This density was expressed as the fraction of the total number of sequences meeting a certain similarity criterion. Therefore this density score will be between 0 (0%, no sequences meeting the similarity criterion) and 1 (100%, all sequences meeting the similarity criterion). We calculated the density at a large number of similarity thresholds between 99% and 70%. Optimal performance was reached at 97%, similarity defined as 1 minus the euclidean distance. Furthermore, this similarity was based on full sequence similarity rather than binding site similarity.

Hence for each sequence, the total number of sequences being 97% similar or more can be between 0.0 (none) and 1.0 (all). We found that in practice the total fraction did not exceed 0.3 (30% of the sequences in the training set 97% similar or more).

### Learning curves

The learning curves provide an estimate for the maximal performance that can be achieved on these datasets, simultaneously they represent external validation. The learning curves show that the models gradually improve when trained on a larger dataset. The results show that PCM is not only able to create models on this data, but also that these models are robust with good validation parameters. The PI model shows the best performance, RMSE = 0.42 log units when trained on 5% of the full set and <0.30 log units when trained on 70% of the dataset. The NNRTI model the worst performance, RMSE = 0.70 log units when trained on 5% of the full set and <0.50 log units when trained on 70% of the dataset (supporting Figure S1).

### Y-Scrambling

Subsequent to learning curve creation, y-scrambled models were created. Here the measured value (*i.e.* Log FC) was randomly permutated over the drug – mutant combination. The rationale being that no correlation should remain as the presence of a certain mutation will no longer be associated with a lower Log FC value but with mixed Log FC values. Supporting Table S2 display the lack of correlation between measured and scrambled values.

Models that were trained on this scrambled set and validated on 30% the data that was kept unscrambled produced very high RMSE values. These values were (in log units); 0.83 (PIs, versus 0.27 for predictive models), 1.10 (NRTIs, versus 0.31 for predictive models) and 1.11 (NNRTIs, versus 0.45 for predictive models). Furthermore, the values for the R_0_^2^ were very low; −0.06 (PIs, versus 0.89 for predictive models), −0.20 (NRTIs, versus 0.75 for predictive models) and −0.21 (NNRTIs, versus 0.79 for predictive models) (Supporting Table S2). Finally the cross validation parameters for the models trained on these scrambled sets demonstrated a lack of correlation; RMSE in log units was highly similar to the external validation; 0.87 (PIs), 1.11 (NRTIs) and 1.12 (NNRTIs). The corresponding correlation coefficient was 0.00 for all three models.

### Model interpretation

To determine the effect of individual residues, for each sequence each residue was mutated back to wild type *in silico* by replacing the descriptors of the mutant amino acid with the descriptors of the corresponding wild type residue as was done previously. [33] Subsequently for all drugs the model prediction on the original mutant sequence was compared with the prediction of the model on the *in silico* changed mutant sequence. The difference was interpreted as the change in pIC_50_ induced by that particular residue, hence providing model interpretability. Changes that led to a 0 value shift in pIC_50_ were removed in the calculation of the average influence of mutations in a particular position, since in all cases this was caused by substitution of identical amino acids.

### Known resistance mutations

Known resistant mutations were retrieved from earlier publications by Johnson *et al.* and compared to our model interpretation. [6], [40] While these papers only mention high impact mutations and are gathered over the full population, they are a good frame of reference for our model interpretation. We used both the most recent publication and one from 2006 as Delavirdine (DLV) has been removed from these overviews due to the fact that it is only used rarely.

### Full sequence resistance mutation identification

Mutations were filtered using the following parameters: have a negative effect on the majority of drugs in a single class; occurrence in the dataset more than once; average Log FC for all compounds >0.4; standard deviation over this average <0.4. This provided us with a number of mutations that lead to an increase in fold change on average, again using literature we discarded any previously known mutations and kept those mutations that were novel. [6], [18], [40]

### Drug specific resistance mutation identification

For all interpretable mutations, the standard deviation was calculated over the average Log FC values per drug within a class. Subsequently all mutations were ranked and the top 30 were retained here. The goal here was to find mutations that have the most diverse effect over the different drugs within a class.

### Benchmark dataset for sequence only model comparison

The dataset we used to compare the performance of PCM models with sequence only models was obtained from Van der Borght *et al.* [39] From the paper the 150 sequences with the largest prediction error were selected per drug class. For mixtures present in this set the average value of each z-scale for each of the present variants at a single position was used as descriptor. Mixtures with more than four possible variants at a single position were discarded leading to a total of 146 NNRTI sequences, 146 NRTI sequences, and 149 PI sequences.

### Stanford University validation set

Prediction of the Stanford University set is of is of particular interest since the correlation between Phenosense and AVG has previously been shown not to be very strong. [63], [64] Yet it should also be noted that the Phenosense assay is in fact more quantitative. AVG measures cell death which can be sensitive to slight differences in the state of the host cells used to grow virus. In particular for mutations M41L, M184V, and T215Y there are differences in Phenosense predictions compared with AVG. [65] While the correlation between Phenosense and VircoTYPE (trained on AVG) is slightly better, there are discrepancies. For instance the resistance profile of d4T and TDF, have been shown to have a Pearson's correlation coefficient <0.8 between the two assays. [51] The reference set was downloaded from the Stanford website (version 5.0, July 30, 2010), from this set the sequences by Virco were removed (as they are presumed to be in the training set, and this would artificially boost the results). The mixtures were removed and this provided us with the following numbers of sequence – compound pairs: 1,252 (NNRTI), 2,190, (NRTI), and 4,356 (PI).

After we predicted the Log FC values for individual drug – mutant pairs using our models, the validation parameters were calculated grouped by: Sequence ID (average and standard deviation), per Isolate (average and standard deviation), per Reference ID (average and standard deviation), per drug (average and standard deviation), per class (total), and per individual drug (total) (Table 5). The predictions per class are also included in Figure 7. Note that the raw data was used and no selection for high quality data was made, furthermore, the data was gathered at different labs, using different assays.

### Clinical Cut-offs

Resistance was also classified using clinical cut-offs (CCOs), here we used the values provided on the Stanford website and the values from AVG were obtained from Virco (supporting Table S10 and supporting Table S11). Subsequently CCP was calculated as a fraction of the total, in addition the fraction of overpredicted clinical response (resistance is predicted higher than measured experimentally) and underpredicted clinical response (resistance is predicted lower than measured experimentally) is included.

## Supporting Information

Dataset S1 **Archive file containing tab delimited text files listing all present mutations for each drug type (NNRTI, NRTI and PI) and their effect as described by our final models.**
(ZIP)

Dataset S2 **Archive file containing an sdf file with the Stanford set formatted to be used in PCM modeling. **This file can be opened using most chemical software packages.
(ZIP)

Figure S1 **Learning curves for each drug class.** The curves serve to give an estimate of the maximal performance possible on this dataset.
(TIF)

Figure S2 **30% validation plots for individual NNRTIs.**
(TIF)

Figure S3 **30% validation plots for individual NRTIs.** Note that a small range appears to translate in a lower R_0_^2^.
(TIF)

Figure S4 **30% validation plots for individual PIs.**
(TIF)

Figure S5 **PCM model performance when predicting Log FC values for unseen mixtures.** The performance is decreasing somewhat compared with the performance on non-mixtures sets, but overall the models are shown to be predictive. Note that the similarity measure shows the large distance between these sequences and the training set as indicated by the small fraction with a (full sequence) similarity larger than 0.97.
(TIF)

Figure S6 **Effects of known RT mutations on NRTI pIC_50_ according to the model.** As was the case with the NNRTIs, the model accurately reproduces resistance of mutations known from literature. Values in the cells represent Log FC. Red colored cells indicate a high Log FC (as shown in the legend), green cells represent a negative Log FC and white cell indicate a Log FC near to 0.
(TIF)

Figure S7 **Effects of known PR mutations on PI pIC_50_ according to the model.** As was the case with the NNRTIs, the model accurately reproduces resistance of mutations known from literature. Values in the cells represent Log FC. Red colored cells indicate a high Log FC (as shown in the legend), green cells represent a negative Log FC and white cell indicate a Log FC near to 0.
(TIF)

Figure S8 **Top 30 mutations that have a diverse effect on NNRTIs pIC_50_.** Note that some values are missing (e.g. the combination T216I – NVP). Values in the cells represent Log FC. Red colored cells indicate a high Log FC (as shown in the legend), green cells represent a negative Log FC and white cell indicate a Log FC near to 0. Note that some values are missing (white cells) this is as these particular mutations did not occur in combination with the drug listed.
(TIF)

Figure S9 **Top 30 mutations that have a diverse effect on NRTIs pIC_50_.** Note that some values are missing (e.g. the combination Q145V – FTC). Values in the cells represent Log FC. Red colored cells indicate a high Log FC (as shown in the legend), green cells represent a negative Log FC and white cell indicate a Log FC near to 0. Note that some values are missing (white cells) this is as these particular mutations did not occur in combination with the drug listed.
(TIF)

Table S1 **Abbreviations for the different drugs.**
(DOC)

Table S2 **Performance of PCM compared to several benchmark approaches.**
(DOC)

Table S3 **Model validation (CCP) on sequences present in the training set (different drugs).**
(DOC)

Table S4 **Model validation (CCP) on sequences not present in the training set.**
(DOC)

Table S5 **Model validation (CCP) LOSO.**
(DOC)

Table S6 **Performance of PCM compared to sequence outliers models (mixtures only).**
(DOC)

Table S7 **Similarity matrix that was used as NNRTI descriptor.**
(DOC)

Table S8 **Similarity matrix that was used as NRTI descriptor.**
(DOC)

Table S9 **Similarity matrix that was used as PI descriptor.**
(DOC)

Table S10 **Clinical cut-off and biological cut-off values used for the NRTIs and NNRTIs.**
(DOC)

Table S11 **Clinical cut-off and biological cut-off values used for the PIs.**
(DOC)
