# Supplementary figures and images for: Significantly Improved HIV Inhibitor Efficacy Prediction Employing Proteochemometric Models Generated From Antivirogram Data
Source: PLoS Comput Biol. 2013 Feb 21;9(2):e1002899. doi: 10.1371/journal.pcbi.1002899 (PMC3578754; doi:10.1371/journal.pcbi.1002899)

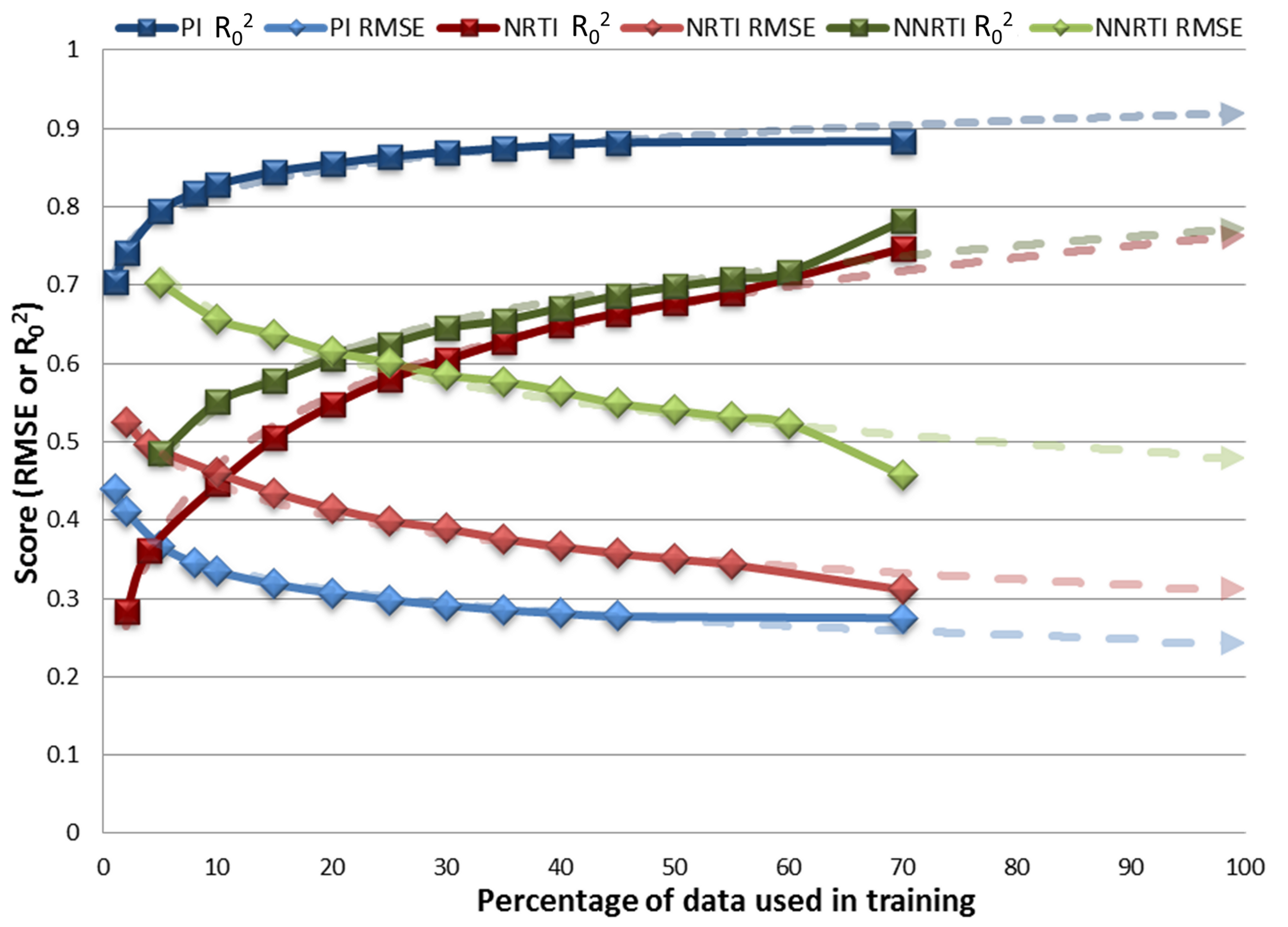

Supplement: Figure S1 — Learning curves for each drug class. The curves serve to give an estimate of the maximal performance possible on this dataset. (TIF) [file pcbi.1002899.s003.tif]

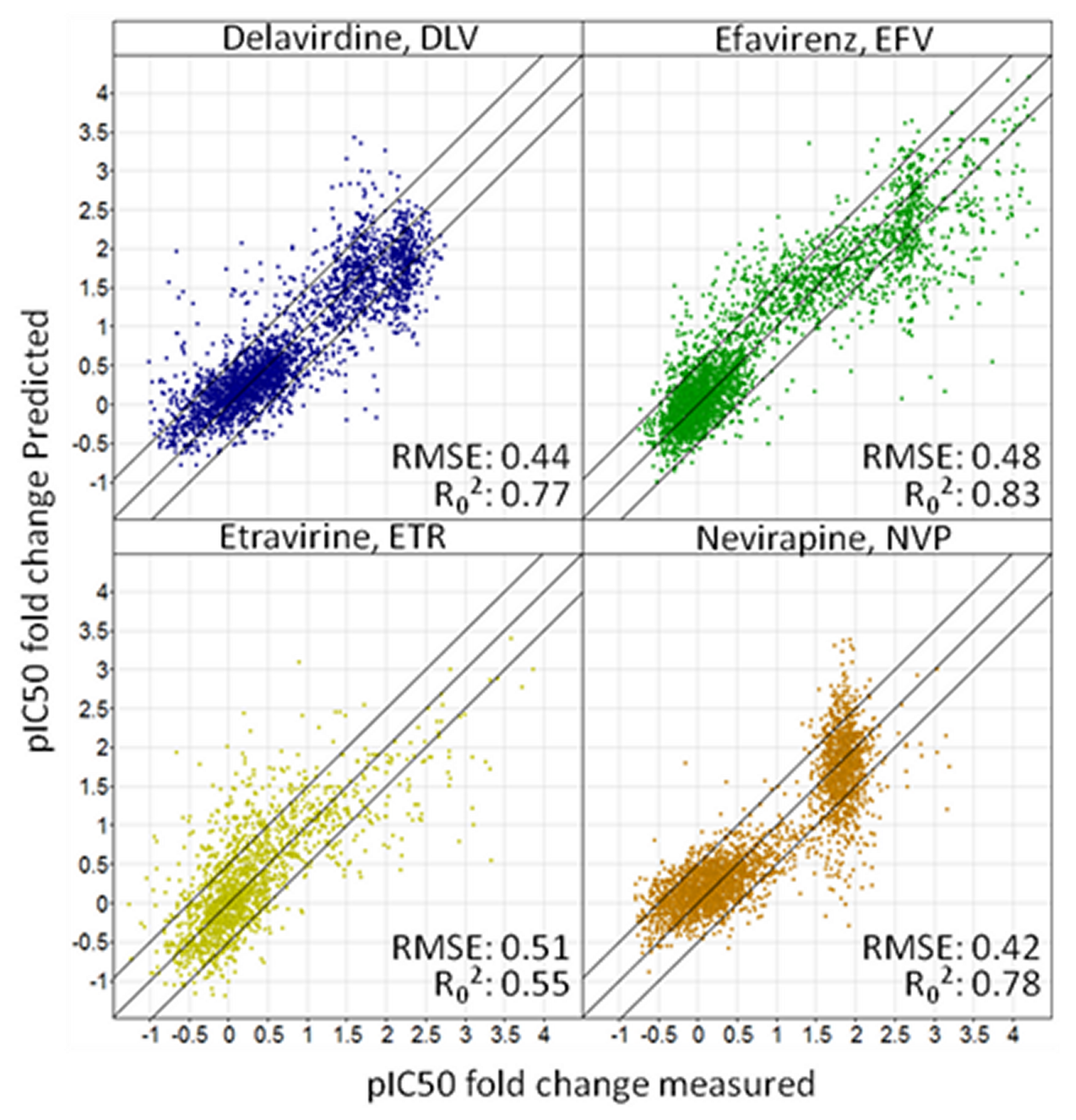

Supplement: Figure S2 — 30% validation plots for individual NNRTIs. (TIF) [file pcbi.1002899.s004.tif]

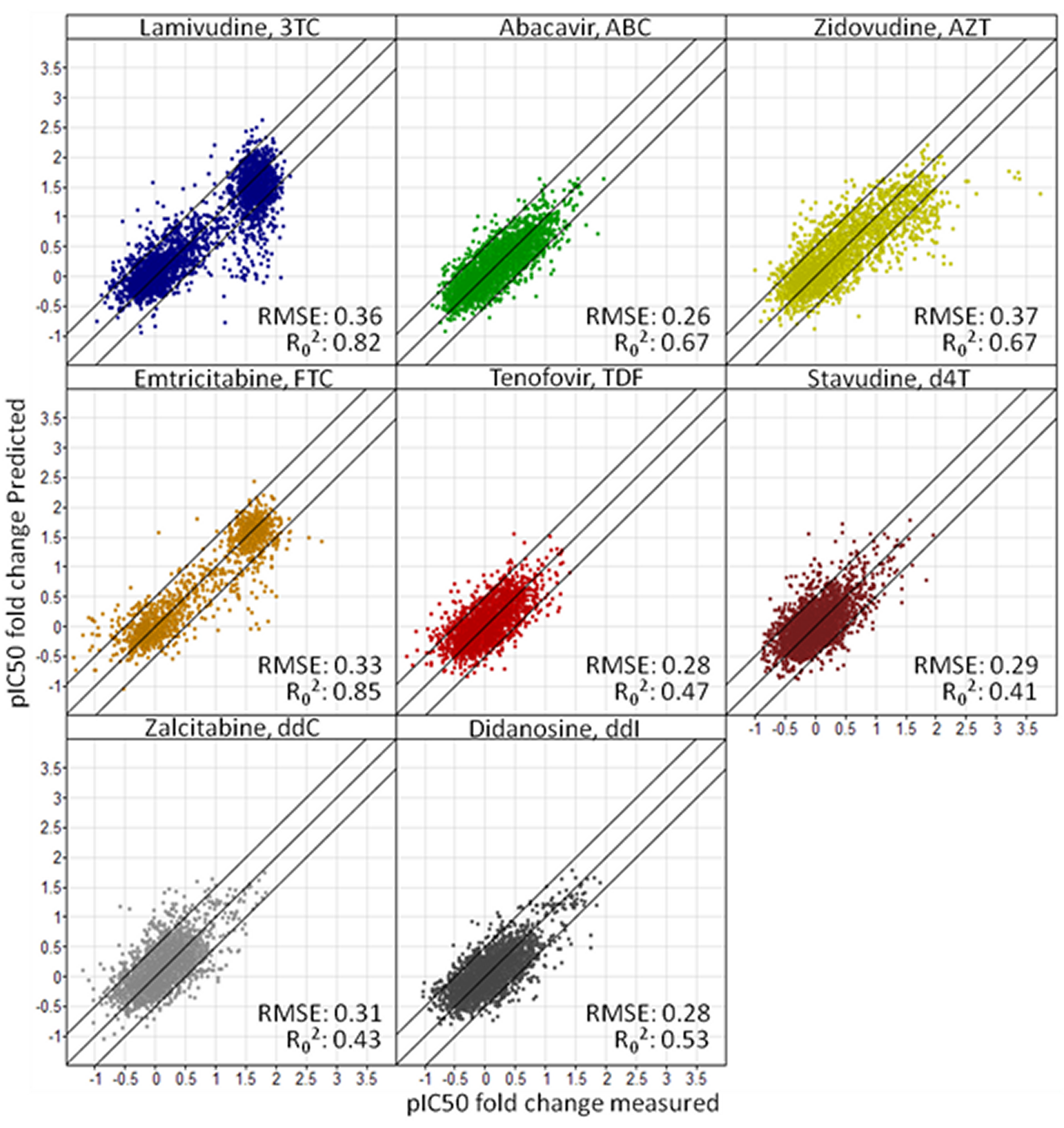

Supplement: Figure S3 — 30% validation plots for individual NRTIs. Note that a small range appears to translate in a lower R0 2. (TIF) [file pcbi.1002899.s005.tif]

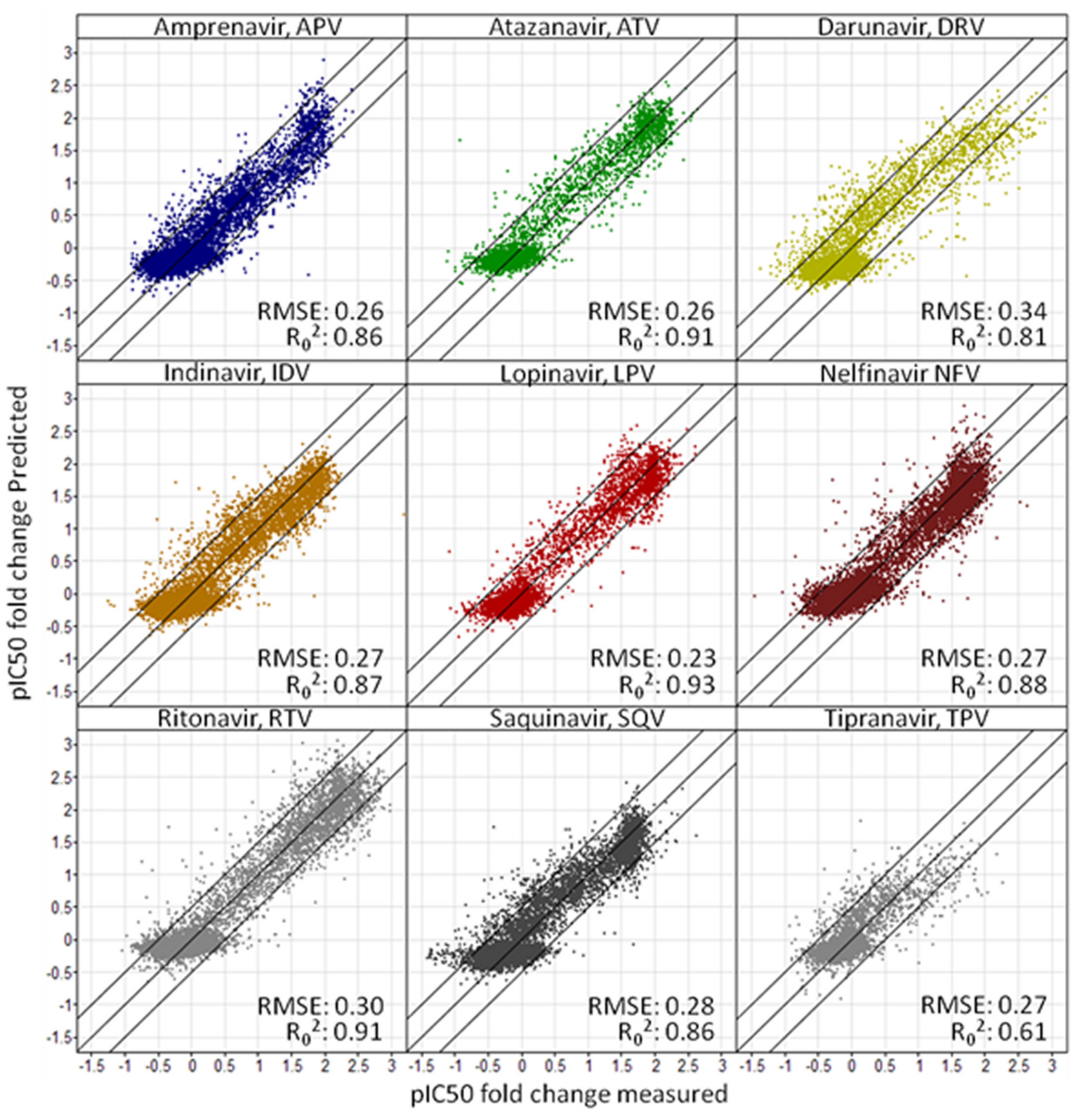

Supplement: Figure S4 — 30% validation plots for individual PIs. (TIF) [file pcbi.1002899.s006.tif]

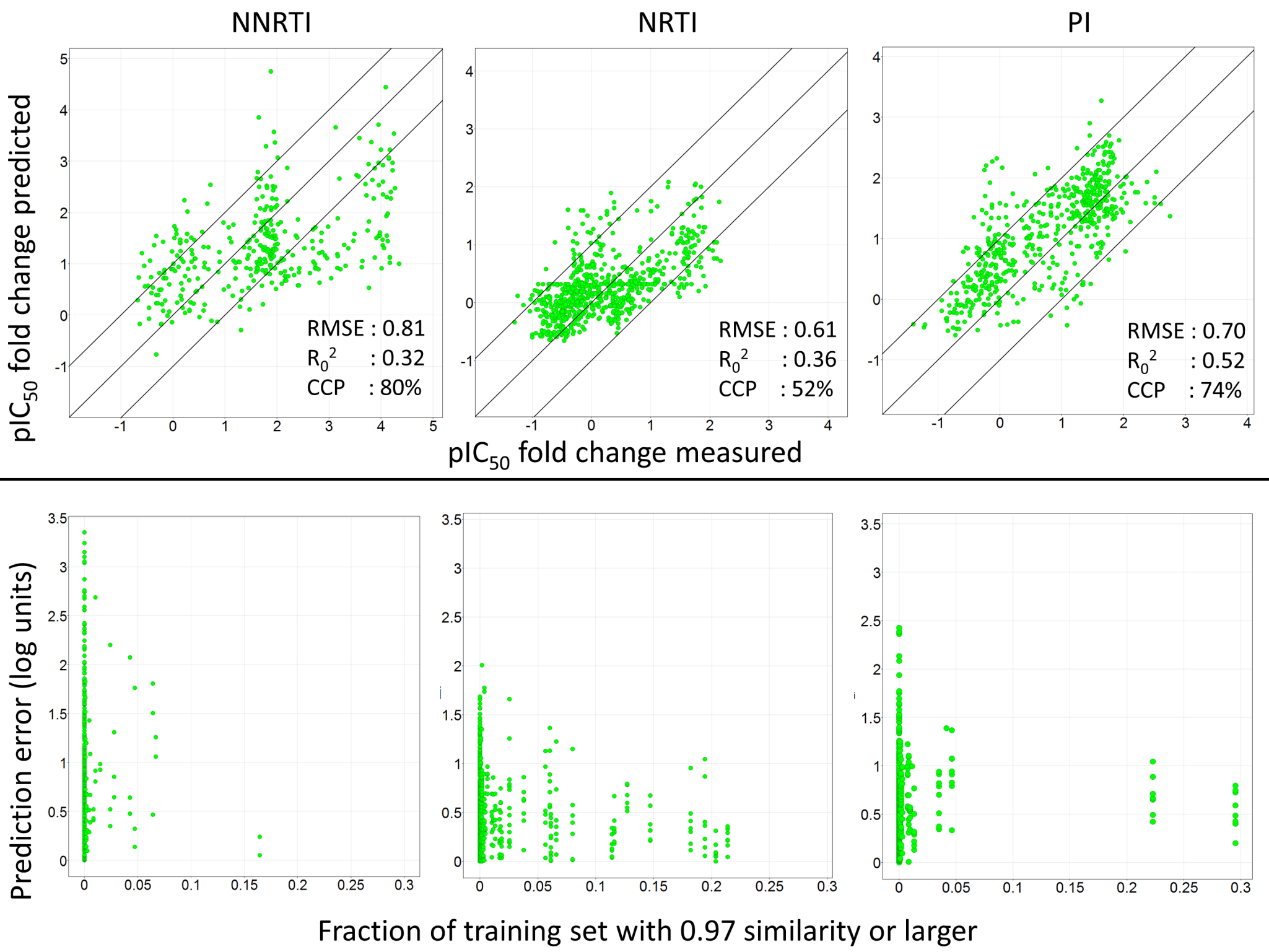

Supplement: Figure S5 — PCM model performance when predicting Log FC values for unseen mixtures. The performance is decreasing somewhat compared with the performance on non-mixtures sets, but overall the models are shown to be predictive. Note that the similarity measure shows the large distance between these sequences and the training set as indicated by the small fraction with a (full sequence) similarity larger than 0.97. (TIF) [file pcbi.1002899.s007.tif]

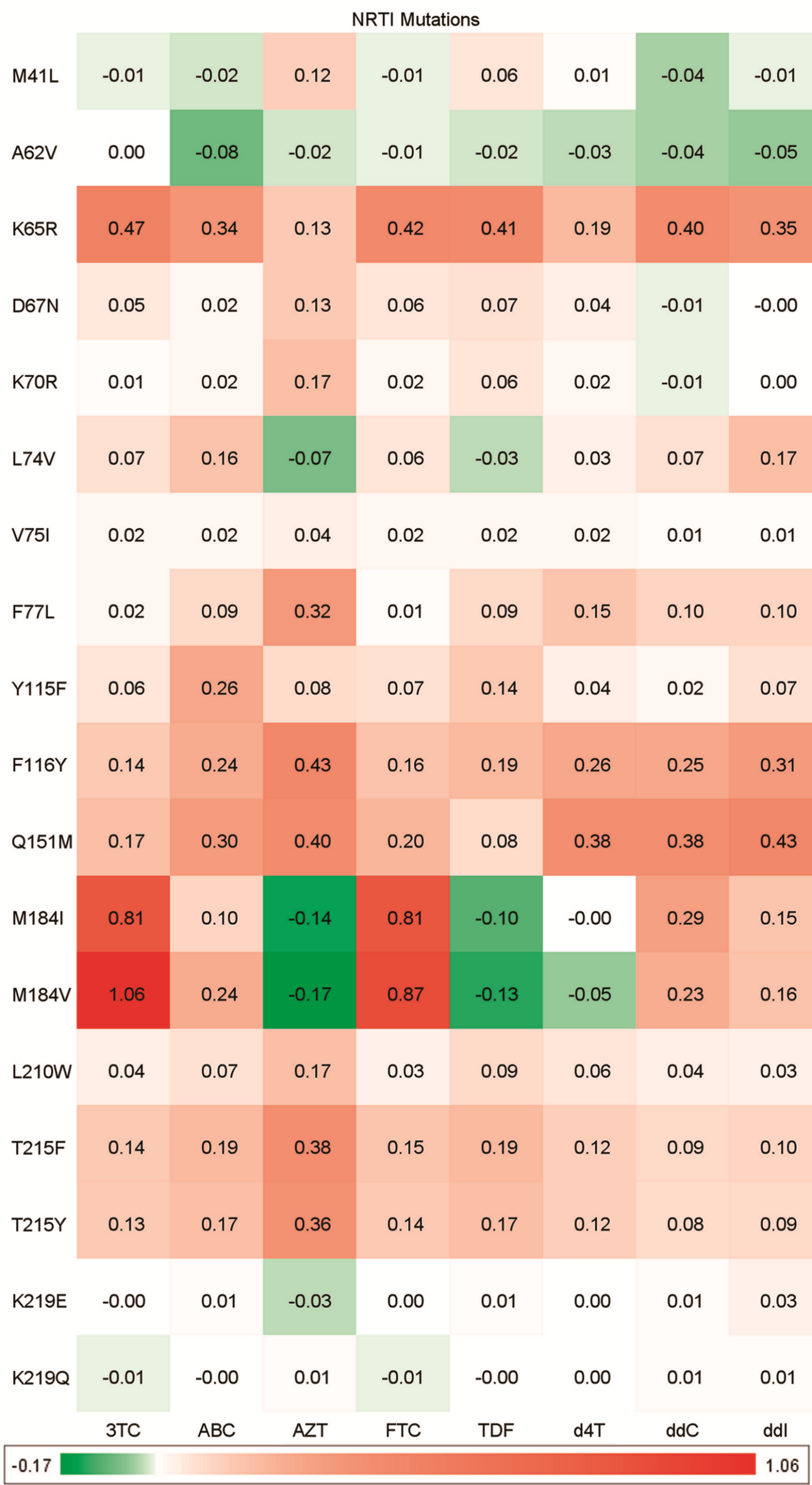

Supplement: Figure S6 — Effects of known RT mutations on NRTI pIC50 according to the model. As was the case with the NNRTIs, the model accurately reproduces resistance of mutations known from literature. Values in the cells represent Log FC. Red colored cells indicate a high Log FC (as shown in the legend), green cells represent a negative Log FC and white cell indicate a Log FC near to 0. (TIF) [file pcbi.1002899.s008.tif]

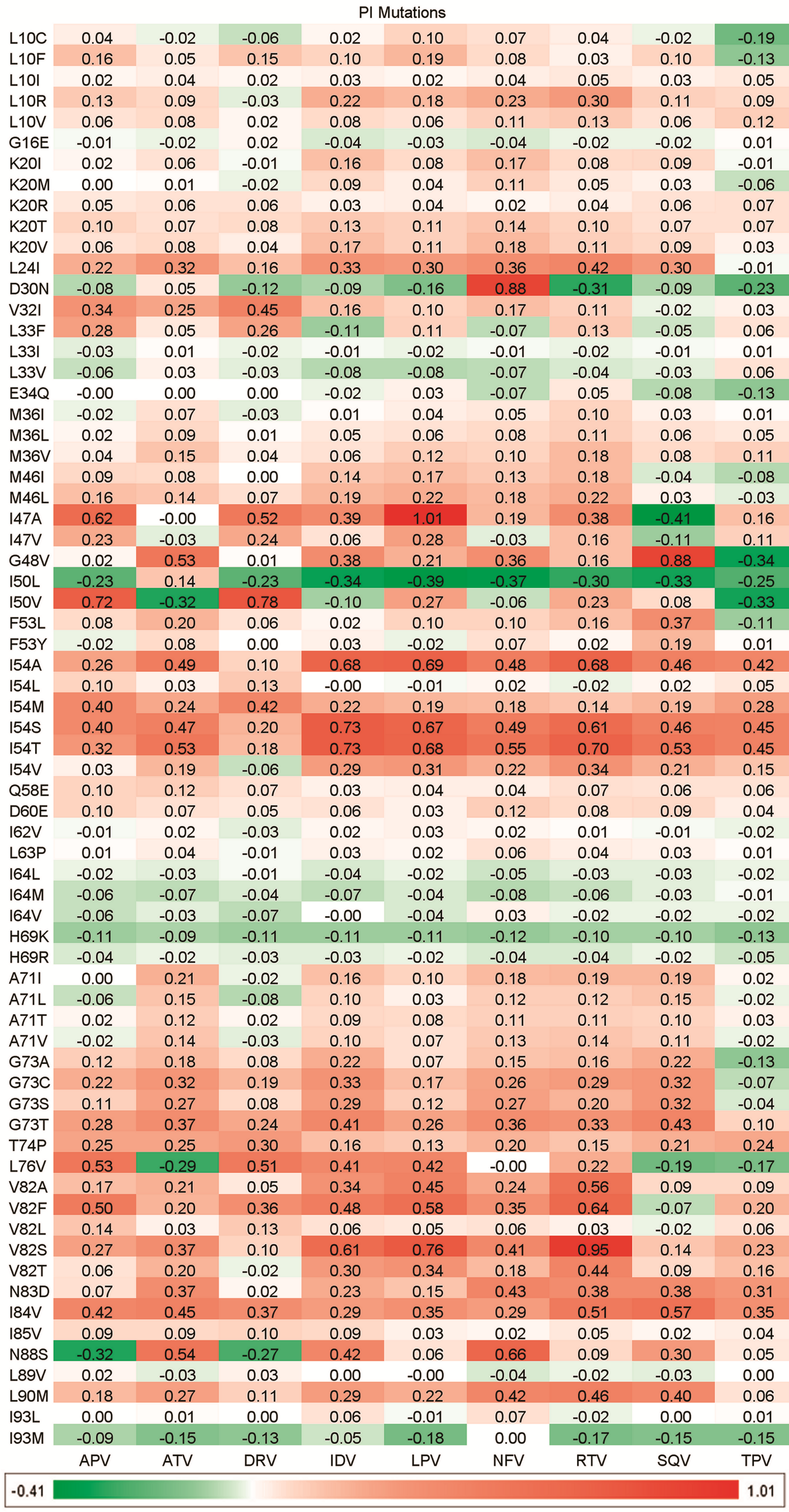

Supplement: Figure S7 — Effects of known PR mutations on PI pIC50 according to the model. As was the case with the NNRTIs, the model accurately reproduces resistance of mutations known from literature. Values in the cells represent Log FC. Red colored cells indicate a high Log FC (as shown in the legend), green cells represent a negative Log FC and white cell indicate a Log FC near to 0. (TIF) [file pcbi.1002899.s009.tif]

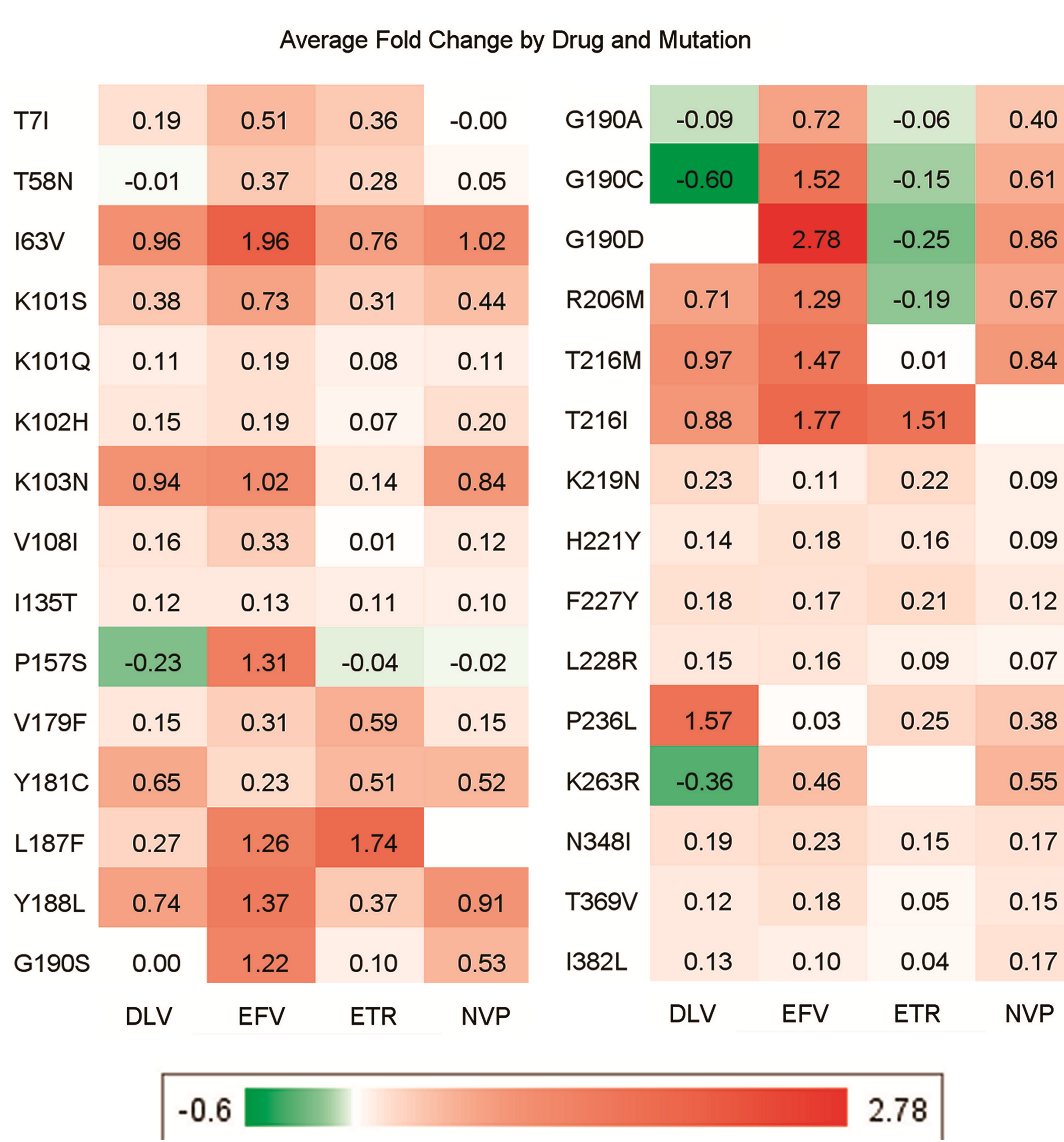

Supplement: Figure S8 — Top 30 mutations that have a diverse effect on NNRTIs pIC50. Note that some values are missing (e.g. the combination T216I – NVP). Values in the cells represent Log FC. Red colored cells indicate a high Log FC (as shown in the legend), green cells represent a negative Log FC and white cell indicate a Log FC near to 0. Note that some values are missing (white cells) this is as these particular mutations did not occur in combination with the drug listed. (TIF) [file pcbi.1002899.s010.tif]

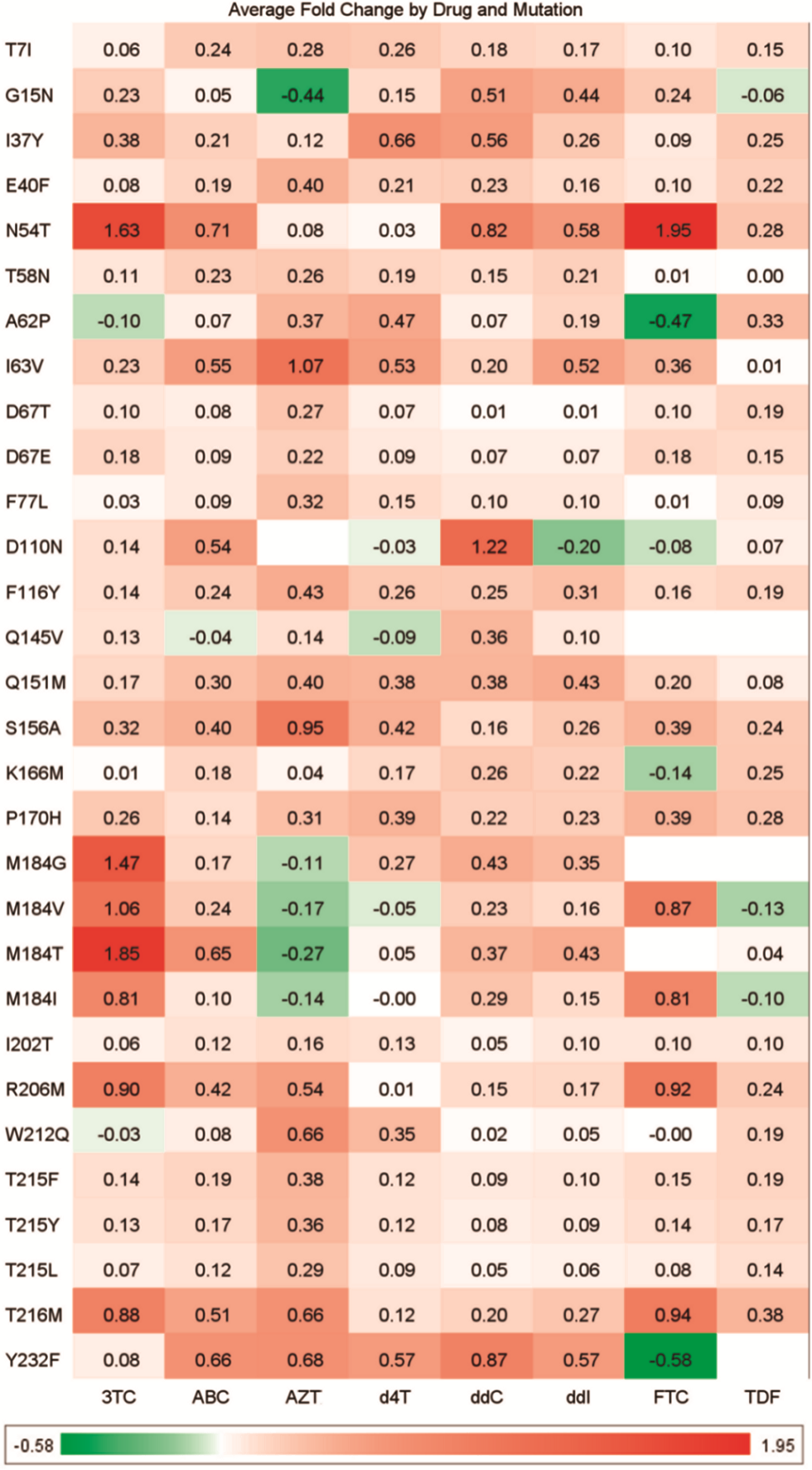

Supplement: Figure S9 — Top 30 mutations that have a diverse effect on NRTIs pIC50. Note that some values are missing (e.g. the combination Q145V – FTC). Values in the cells represent Log FC. Red colored cells indicate a high Log FC (as shown in the legend), green cells represent a negative Log FC and white cell indicate a Log FC near to 0. Note that some values are missing (white cells) this is as these particular mutations did not occur in combination with the drug listed. (TIF) [file pcbi.1002899.s011.tif]
