# Supplementary material for: Significantly Improved HIV Inhibitor Efficacy Prediction Employing Proteochemometric Models Generated From Antivirogram Data
Source: PLoS Comput Biol. 2013 Feb 21;9(2):e1002899. doi: 10.1371/journal.pcbi.1002899 (PMC3578754; doi:10.1371/journal.pcbi.1002899)
Supplement: Table S1 — Abbreviations for the different drugs. (DOC) [file pcbi.1002899.s012.doc]

# Table S1: Abbreviations for the different drugs

| Name | Drug | Class |
| --- | --- | --- |
| DLV | Delavirdine | NNRTI |
| EFV | Efavirenz | NNRTI |
| ETR | Etravirine | NNRTI |
| NVP | Nevirapine | NNRTI |
| 3TC | Lamivudine | NRTI |
| ABC | Abacavir | NRTI |
| AZT | Zidovudine | NRTI |
| D4T | Stavudine | NRTI |
| DDC | Zalcitabine | NRTI |
| DDI | Didanosine | NRTI |
| TDF | Tenofovir | NRTI |
| FTC | Emtricitabine | NRTI |
| APV | Amprenavir / Fosamprenavir | PI |
| ATV | Atazanavir | PI |
| DRV | Darunavir | PI |
| IDV | Indinavir | PI |
| LPV | Lopinavir | PI |
| NFV | Nelfinavir | PI |
| RTV | Ritonavir | PI |
| SQV | Saquinavir | PI |
| TPV | Tipranavir | PI |

Shown are the abbreviations we used to refer to a drug, also shown is the class the individual drug belong to.
