# Supplementary material for: Significantly Improved HIV Inhibitor Efficacy Prediction Employing Proteochemometric Models Generated From Antivirogram Data
Source: PLoS Comput Biol. 2013 Feb 21;9(2):e1002899. doi: 10.1371/journal.pcbi.1002899 (PMC3578754; doi:10.1371/journal.pcbi.1002899)
Supplement: Table S2 — Performance of PCM compared to several benchmark approaches. (DOC) [file pcbi.1002899.s013.doc]

# Table S2: Performance of PCM compared to several benchmark approaches

|  | NNRTI RMSE | NNRTI  R_0_^2^ | NRTI  RMSE | NRTI  R_0_^2^ | PI  RMSE | PI  R_0_^2^ |
| --- | --- | --- | --- | --- | --- | --- |
| PCM | 0.45 | 0.79 | 0.31 | 0.75 | 0.27 | 0.89 |
| Sequence only | 0.50 | 0.74 | 0.35 | 0.69 | 0.30 | 0.86 |
| Resistance Scaling | 0.62 | 0.54 | 0.59 | 0.20 | 0.46 | 0.69 |
| Y-Scambling | 1.11 | 0.00 | 1.10 | 0.00 | 0.83 | 0.06 |
|  |  |  |  |  |  |  |
| PCM (Van der Borght set) | 0.85 | 0.28 | 0.53 | 0.51 | 0.65 | 0.54 |
| PCM (Van der Borght set Mixtures) | 0.81 | 0.32 | 0.61 | 0.36 | 0.70 | 0.52 |
| Performance Van der Borght *et al.* | 1.30 | 0.00 | 0.68 | 0.27 | 0.75 | 0.56 |
| Performance Van der Borght *et al.*(Mixtures) | 1.34 | 0.00 | 0.68 | 0.30 | 0.75 | 0.58 |
|  |  |  |  |  |  |  |
| PCM (Stanford set) | 0.68 | 0.65 | 0.61 | 0.39 | 0.44 | 0.75 |

Different benchmark techniques are compared to our PCM models per HIV drug class. Negative correlation coefficients were annotated as 0.00. Also shown is the performance of our models validated on a previously unseen dataset by Van der Borght *et al.* and on the Stanford set.
