# Supplementary material for: Significantly Improved HIV Inhibitor Efficacy Prediction Employing Proteochemometric Models Generated From Antivirogram Data
Source: PLoS Comput Biol. 2013 Feb 21;9(2):e1002899. doi: 10.1371/journal.pcbi.1002899 (PMC3578754; doi:10.1371/journal.pcbi.1002899)
Supplement: Table S3 — Model validation (CCP) on sequences present in the training set (different drugs). (DOC) [file pcbi.1002899.s014.doc]

# Table S3: Model validation (CCP) on sequences present in the training set (different drugs).

| Correctly Classified Percentage | Overpredicted  Percentage | Underpredicted  Percentage | Class/Drug |
| --- | --- | --- | --- |
| 0.93 | 0.03 | 0.04 | PI Class |
| 0.94 | 0.03 | 0.03 | APV |
| 0.96 | 0.02 | 0.01 | ATV |
| 0.96 | 0.03 | 0.02 | DRV |
| 0.84 | 0.03 | 0.13 | IDV |
| 0.97 | 0.02 | 0.01 | LPV |
| 0.94 | 0.02 | 0.04 | NFV |
| 0.97 | 0.01 | 0.02 | RTV |
| 0.95 | 0.04 | 0.01 | SQV |
| 0.90 | 0.07 | 0.04 | TPV |
| 0.93 | 0.03 | 0.04 | NNRTI Class |
| 0.87 | 0.05 | 0.07 | DLV |
| 1.00 | 0.00 | 0.00 | EFV |
| 0.85 | 0.12 | 0.03 | ETR |
| 0.95 | 0.01 | 0.04 | NVP |
| 0.80 | 0.10 | 0.10 | NRTI Class |
| 0.91 | 0.05 | 0.04 | 3TC |
| 0.82 | 0.09 | 0.09 | ABC |
| 0.81 | 0.09 | 0.09 | AZT |
| 0.70 | 0.15 | 0.15 | d4T |
| 0.76 | 0.12 | 0.12 | ddC |
| 0.76 | 0.12 | 0.13 | ddI |
| 0.96 | 0.02 | 0.03 | FTC |
| 0.75 | 0.12 | 0.12 | TDF |
| **0.95** | **0.02** | **0.03** | **Overall** |

For four drugs (APV, RTV, DLV, DDC) no Virco cut-off was available, here the Stanford cut off was used
