# Supplementary material for: Significantly Improved HIV Inhibitor Efficacy Prediction Employing Proteochemometric Models Generated From Antivirogram Data
Source: PLoS Comput Biol. 2013 Feb 21;9(2):e1002899. doi: 10.1371/journal.pcbi.1002899 (PMC3578754; doi:10.1371/journal.pcbi.1002899)
Supplement: Table S4 — Model validation (CCP) on sequences not present in the training set. (DOC) [file pcbi.1002899.s015.doc]

# Table S4: Model validation (CCP) on sequences not present in the training set.

| Correctly Classified Percentage | Overpredicted  Percentage | Underpredicted  Percentage | Class/Drug |
| --- | --- | --- | --- |
| 0.89 | 0.04 | 0.07 | PI Class |
| 0.88 | 0.00 | 0.12 | APV |
| 0.93 | 0.00 | 0.07 | ATV |
| 0.88 | 0.05 | 0.07 | DRV |
| 0.74 | 0.03 | 0.24 | IDV |
| 1.00 | 0.00 | 0.00 | LPV |
| 0.95 | 0.00 | 0.05 | NFV |
| 0.98 | 0.02 | 0.00 | RTV |
| 0.86 | 0.11 | 0.03 | SQV |
| 1.00 | 0.00 | 0.00 | TPV |
| 0.91 | 0.05 | 0.04 | NNRTI Class |
| 0.82 | 0.06 | 0.11 | DLV |
| 1.00 | 0.00 | 0.00 | EFV |
| 0.83 | 0.16 | 0.01 | ETR |
| 0.92 | 0.04 | 0.04 | NVP |
| 0.68 | 0.14 | 0.17 | NRTI Class |
| 0.80 | 0.20 | 0.00 | AZT |
| 0.60 | 0.20 | 0.20 | 3TC |
| 0.50 | 0.25 | 0.25 | ABC |
| 0.40 | 0.20 | 0.40 | d4T |
| 0.71 | 0.14 | 0.14 | ddC |
| 0.60 | 0.40 | 0.00 | ddI |
| 0.82 | 0.00 | 0.18 | FTC |
| 0.00 | 0.00 | 1.00 | TDF |
| **0.91** | **0.04** | **0.05** | **Overall** |

For four drugs (APV, RTV, DLV, DDC) no Virco cut-off was available, here the Stanford cut off was used
