# Supplementary material for: Significantly Improved HIV Inhibitor Efficacy Prediction Employing Proteochemometric Models Generated From Antivirogram Data
Source: PLoS Comput Biol. 2013 Feb 21;9(2):e1002899. doi: 10.1371/journal.pcbi.1002899 (PMC3578754; doi:10.1371/journal.pcbi.1002899)
Supplement: Table S6 — Performance of PCM compared to sequence outliers models (mixtures only). (DOC) [file pcbi.1002899.s017.doc]

# Table S6: Performance of PCM compared to sequence outliers models (mixtures only).

| RMSE (Log units) | R_0_^2^ | RMSE Sequence only (Log Units) | R_0_^2^ Sequence only | Grouping |
| --- | --- | --- | --- | --- |
| 0.73 (± 0.26) | 0.33 (±0.20) | 0.80 (± 0.29) | 0.27 (±0.33) | Drug (average) |
| *0.70* | *0.52* | *0.75* | *0.58* | *PI (Class)* |
| 0.68 | 0.60 | 0.68 | 0.67 | APV |
| 0.77 | 0.50 | 0.86 | 0.49 | ATV |
| 0.70 | 0.59 | 0.70 | 0.64 | DRV |
| 0.71 | 0.48 | 0.76 | 0.60 | IDV |
| 0.74 | 0.57 | 0.87 | 0.65 | LPV |
| 0.71 | 0.45 | 0.76 | 0.47 | NFV |
| 0.71 | 0.43 | 0.80 | 0.50 | SQV |
| 0.57 | 0.27 | 0.54 | 0.42 | TPV |
| *0.81* | *0.32* | *1.3* | *0.00* | *NNRTI (Class)* |
| 1.0 | 0.33 | 1.1 | 0.14 | ETR |
| 1.7 | 0.01 | 1.9 | 0.00 | EFV |
| 0.77 | 0.00 | 0.88 | 0.00 | NVP |
| *0.61* | *0.36* | *0.68* | *0.30* | *NRTI (Class)* |
| 0.77 | 0.33 | 0.85 | 0.32 | 3TC |
| 0.48 | 0.33 | 0.54 | 0.19 | ABC |
| 0.67 | 0.33 | 0.76 | 0.19 | AZT |
| 0.53 | 0.16 | 0.56 | 0.05 | D4T |
| 0.49 | 0.21 | 0.52 | 0.12 | DDI |
| 0.76 | 0.29 | 0.83 | 0.30 | FTC |
| 0.50 | 0.19 | 0.56 | 0.02 | TDF |
| ***0.73*** | ***0.33*** | ***0.80*** | ***0.27*** | ***Overall*** |

Validation parameters were calculated using different forms of grouping to give an unbiased error estimate. The table shows that even when predicting the Log FC for mixtures, which were not included in the training set, our PCM models perform better than sequence only models. This is indicated by the regression validation parameters RMSE and R_0_^2^. While it should be noted that for the PIs, the sequence only models tend to have a slightly higher R_0_^2^, they also have a higher RMSE.
