# Supplementary material for: Significantly Improved HIV Inhibitor Efficacy Prediction Employing Proteochemometric Models Generated From Antivirogram Data
Source: PLoS Comput Biol. 2013 Feb 21;9(2):e1002899. doi: 10.1371/journal.pcbi.1002899 (PMC3578754; doi:10.1371/journal.pcbi.1002899)
Supplement: Table S7 — Similarity matrix that was used as NNRTI descriptor. (DOC) [file pcbi.1002899.s018.doc]

# Table S7: Similarity Matrix that was used as NNRTI descriptor.

| Drug | NVP | DLV | EFV | ETR | Average |
| --- | --- | --- | --- | --- | --- |
| NVP | 1.00 | 0.31 | 0.16 | 0.18 | 0.41 |
| DLV | 0.23 | 1.00 | 0.12 | 0.15 | 0.38 |
| EFV | 0.16 | 0.17 | 1.00 | 0.11 | 0.36 |
| ETR | 0.16 | 0.18 | 0.10 | 1.00 | 0.36 |
| Average | 0.39 | 0.42 | 0.35 | 0.36 |  |

Each row (excluding the drug name) formed the descriptor for the drug listed in the first column. On average the similarity between the different NNRTIs is the lowest compared with the NRTIs and PIs. Also shown is the average similarity of each compound to the rest of the compounds.
