# Supplementary material for: Significantly Improved HIV Inhibitor Efficacy Prediction Employing Proteochemometric Models Generated From Antivirogram Data
Source: PLoS Comput Biol. 2013 Feb 21;9(2):e1002899. doi: 10.1371/journal.pcbi.1002899 (PMC3578754; doi:10.1371/journal.pcbi.1002899)
Supplement: Table S9 — Similarity matrix that was used as PI descriptor. (DOC) [file pcbi.1002899.s020.doc]

# Table S9: Similarity Matrix that was used as PI descriptor.

| Drug | SQV | RTV | IDV | NFV | LPV | ATV | APV | TPV | DRV | Average |
| --- | --- | --- | --- | --- | --- | --- | --- | --- | --- | --- |
| SQV | 1.00 | 0.32 | 0.33 | 0.49 | 0.29 | 0.32 | 0.28 | 0.20 | 0.31 | 0.39 |
| RTV | 0.32 | 1.00 | 0.29 | 0.21 | 0.35 | 0.32 | 0.33 | 0.21 | 0.32 | 0.37 |
| IDV | 0.35 | 0.31 | 1.00 | 0.32 | 0.28 | 0.31 | 0.28 | 0.24 | 0.30 | 0.38 |
| NFV | 0.57 | 0.25 | 0.35 | 1.00 | 0.26 | 0.26 | 0.26 | 0.25 | 0.28 | 0.39 |
| LPV | 0.33 | 0.40 | 0.30 | 0.25 | 1.00 | 0.32 | 0.31 | 0.26 | 0.32 | 0.39 |
| APV | 0.36 | 0.42 | 0.35 | 0.28 | 0.35 | 0.36 | 1.00 | 0.22 | 0.79 | 0.46 |
| ATV | 0.37 | 0.37 | 0.33 | 0.25 | 0.32 | 1.00 | 0.32 | 0.23 | 0.35 | 0.39 |
| TPV | 0.22 | 0.24 | 0.25 | 0.23 | 0.25 | 0.22 | 0.19 | 1.00 | 0.22 | 0.31 |
| DRV | 0.37 | 0.38 | 0.33 | 0.28 | 0.33 | 0.37 | 0.73 | 0.24 | 1.00 | 0.45 |
| Average | 0.43 | 0.41 | 0.39 | 0.37 | 0.38 | 0.39 | 0.41 | 0.32 | 0.43 |  |

Each row (excluding the drug name) formed the descriptor for the drug listed in the first column. On average the similarity between the different PIs is lower than the similarity between NRTIs and higher than the similarity of NNRTIs. Also shown is the average similarity of each compound to the rest of the compounds.
