# Supplementary material for: Significantly Improved HIV Inhibitor Efficacy Prediction Employing Proteochemometric Models Generated From Antivirogram Data
Source: PLoS Comput Biol. 2013 Feb 21;9(2):e1002899. doi: 10.1371/journal.pcbi.1002899 (PMC3578754; doi:10.1371/journal.pcbi.1002899)
Supplement: Table S10 — Clinical cut-off and biological cut-off values used for the NRTIs and NNRTIs. (DOC) [file pcbi.1002899.s021.doc]

# Table S10: Clinical cut-off and biological cut-off values used for the NRTIs and NNRTIs.

|  |  | Fold Change Virco TYPE | | |  | Log Fold Change Virco TYPE | | | | |  | Fold Change Phenosense | | |  | Log Fold Change Phenosense | | |
| --- | --- | --- | --- | --- | --- | --- | --- | --- | --- | --- | --- | --- | --- | --- | --- | --- | --- | --- |
| Drug | Code | cco1 | cco2 | bco |  | cco1 | | cco2 | | bco |  | cco1 | cco2 | bco |  | cco1 | cco2 | bco |
| Zidovudine | AZT | 1.5 | 11.4 |  |  | 0.18 | | 1.06 | |  |  | 3 | 25 |  |  | 0.48 | 1.40 |  |
| Lamivudine | 3TC | 2.1 | 4.6 |  |  | 0.32 | | 0.66 | |  |  | 3 | 25 |  |  | 0.48 | 1.40 |  |
| Didanosine | ddI | 0.9 | 2.6 |  |  | -0.05 | | 0.41 | |  |  | 1.5 | 3 |  |  | 0.18 | 0.48 |  |
| Zalcitabine | ddC |  |  |  |  |  | |  | |  |  | 1.5 | 3 |  |  | 0.18 | 0.48 |  |
| Stavudine | d4T | 1 | 2.3 |  |  | 0.00 | | 0.36 | |  |  | 1.5 | 3 |  |  | 0.18 | 0.48 |  |
| Abacavir | ABC | 0.9 | 3.5 |  |  | -0.05 | | 0.54 | |  |  | 2 | 6 |  |  | 0.30 | 0.78 |  |
| Emtricitabine | FTC |  |  | 3.1 |  |  | |  | | 0.49 |  |  |  |  |  |  |  |  |
| Tenofovir | TDF | 1 | 2.3 |  |  | 0.00 | | 0.36 | |  |  | 1.5 | 3 |  |  | 0.18 | 0.48 |  |
| Nevirapine | NVP |  |  | 6 |  |  |  | | 0.78 | |  | 3 | 25 |  |  | 0.48 | 1.40 |  |
| Delavirdine | DLV |  |  |  |  |  |  | |  | |  | 3 | 25 |  |  | 0.48 | 1.40 |  |
| Efavirenz | EFV |  |  | 3.3 |  |  |  | | 0.52 | |  | 3 | 25 |  |  | 0.48 | 1.40 |  |
| Etravirine | ETR | 3.2 | 27.6 |  |  | 0.51 | 1.44 | |  | |  |  |  |  |  |  |  |  |
|  |  |  |  |  |  |  | |  | |  |  |  |  |  |  |  |  |  |

Cco1 represents a reduced response, cco 2 represents a minimal response. Bco represents a biological cut off (deemed resistant) when no cco was available.
