# Supplementary material for: Significantly Improved HIV Inhibitor Efficacy Prediction Employing Proteochemometric Models Generated From Antivirogram Data
Source: PLoS Comput Biol. 2013 Feb 21;9(2):e1002899. doi: 10.1371/journal.pcbi.1002899 (PMC3578754; doi:10.1371/journal.pcbi.1002899)
Supplement: Table S11 — Clinical cut-off and biological cut-off values used for the PIs. (DOC) [file pcbi.1002899.s022.doc]

# Table S11: Clinical cut-off and biological cut-off values used for the PIs.

|  |  | Fold Change Virco TYPE | | |  | Log Fold Change Virco TYPE | | |  | Fold Change Phenosense | | |  | Log Fold Change Phenosense | | |
| --- | --- | --- | --- | --- | --- | --- | --- | --- | --- | --- | --- | --- | --- | --- | --- | --- |
| Name | Drug | cco1 | cco2 | bco |  | cco1 | cco2 | bco |  | cco1 | cco2 | bco |  | cco1 | cco2 | bco |
| Indinavir | IDV | 1 | 5.4 |  |  | 0.00 | 0.73 |  |  | 3 | 20 |  |  | 0.48 | 1.30 |  |
| Indinavir/r | IDV/r | 2.3 | 27.2 |  |  | 0.36 | 1.43 |  |  |  |  |  |  |  |  |  |
| Ritonavir | RTV |  |  |  |  |  |  |  |  | 3 | 20 |  |  | 0.48 | 1.30 |  |
| Nelfinavir | NFV | 2.2 | 9.4 |  |  | 0.34 | 0.97 |  |  | 3 | 20 |  |  | 0.48 | 1.30 |  |
| Saquinavir | SQV |  |  |  |  |  |  |  |  | 3 | 20 |  |  | 0.48 | 1.30 |  |
| Saquinavir/r | SQV/r | 3.1 | 22.6 |  |  | 0.49 | 1.35 |  |  |  |  |  |  |  |  |  |
| Amprenavir | AMP |  |  |  |  |  |  |  |  | 3 | 20 |  |  | 0.48 | 1.30 |  |
| Fosamprenavir/r | FPV/r | 1.5 | 19.5 |  |  | 0.18 | 1.29 |  |  |  |  |  |  |  |  |  |
| Lopinavir | LPV |  |  |  |  |  |  |  |  | 3 | 20 |  |  | 0.48 | 1.30 |  |
| Lopinavir/r | LPV/r | 6.1 | 51.2 |  |  | 0.79 | 1.71 |  |  |  |  |  |  |  |  |  |
| Atazanavir | ATV |  |  |  |  |  |  |  |  | 3 | 20 |  |  | 0.48 | 1.30 |  |
| Atazanavir/r | ATV/r | 2.5 | 32.5 |  |  | 0.40 | 1.51 |  |  |  |  |  |  |  |  |  |
| Tipranavir | TPV |  |  |  |  |  |  |  |  |  |  |  |  |  |  |  |
| Tipranavir/r | TPV/r | 1.5 | 7 |  |  | 0.18 | 0.85 |  |  |  |  |  |  |  |  |  |
| Darunavir | DRV |  |  |  |  |  |  |  |  |  |  |  |  |  |  |  |
| Darunavir /r | DRV/r | 10 | 106.9 |  |  | 1.00 | 2.03 |  |  |  |  |  |  |  |  |  |

Cco1 represents a reduced response, cco 2 represents a minimal response. Bco represents a biological cut off (deemed resistant) when no cco was available. When available the non-Ritonavir boosted value was used, if this was not available the value for Ritonavir boosted cco values was used (indicated by /r in the name column)
